# Therapeutic Efficacy and Safety of Intraperitoneally Administered ^211^At-Labeled Gold Nanoparticles for Peritoneally Disseminated Malignancies

**DOI:** 10.3390/pharmaceutics18091102

**Published:** 2026-09-02

**Authors:** Hiroki Kato, Xuhao Huang, Erina Hilmayanti, Yuichiro Kadonaga, Kazuhiro Ooe, Masashi Murakami, Kazuya Kabayama, Kazuko Kaneda-Nakashima, Atsushi Toyoshima, Xiaojie Yin, Hiromitsu Haba, Koichi Fukase

**Affiliations:** 1 Institute for Radiation Sciences, The University of Osaka, 2-4, Yamadaoka, Suita 565-0871, Osaka, Japan; huang@irs.osaka-u.ac.jp (X.H.); kadonaga.yuuichirou.med@osaka-u.ac.jp (Y.K.); ooe@rirc.osaka-u.ac.jp (K.O.); murakami@irs.osaka-u.ac.jp (M.M.); kaba@irs.osaka-u.ac.jp (K.K.); kkaneda@irs.osaka-u.ac.jp (K.K.-N.); toyo@irs.osaka-u.ac.jp (A.T.); fukase.koichi.irs@osaka-u.ac.jp (K.F.); 2 Department of Chemistry, Graduate School of Science, The University of Osaka, Toyonaka 560-0043, Osaka, Japan; erina.hilmayanti99@gmail.com; 3 Nishina Center for Accelerator-Based Science, RIKEN, Wako 351-0198, Saitama, Japan; yinxj04@impcas.ac.cn (X.Y.); haba@riken.jp (H.H.)

**Keywords:** astatine-211, alpha emitters, gold nanoparticles, radiolabeling, cancer therapy

## Abstract

**Background/Objectives**: Peritoneal dissemination of malignancies leads to poor prognoses, and no effective treatment currently exists. The difficulty of treating such malignancies is likely because systemically administered drugs cannot easily target malignant cells in the abdominal cavity. High intraperitoneal drug retention, non-toxicity towards normal tissues, and successful targeting of malignant cells are important for an effective therapy. The aim of this study was to evaluate an intraperitoneally administered astatine-labeled, integrin-targeted nanodrug, mPEG(Mn:350)-S-AuNP[^211^At]-*c*[RGDfK(C)] ([^211^At]AuNP@PEG/RGD), with respect to its kinetics, therapeutic efficacy, and safety. **Methods**: C6 rat glioma cells (10^7^), and BxPC3 (10^7^) and PANC-1 (10^7^) human pancreatic cancer cells were seeded intraperitoneally into nude mice, and [^211^At]AuNP@PEG/RGD (0.979 ± 0.194 MBq for C6 models (*n* = 3), 1.139 ± 0.035 MBq for BxPC3 models (*n* = 10), and 1.308 ± 0.039 MBq for PANC-1 models (*n* = 10) per mouse) or saline were intraperitoneally administered 4–7 days later. Cytotoxicity against malignant cells, pharmacokinetics after administration, therapeutic efficacy, and safety in abdominal organs were evaluated. **Results**: Intraperitoneally administered [^211^At]AuNP@PEG/RGD accumulated exclusively in the peritoneal cavity for a long period of time and showed minimal systemic diffusion through the blood. In the C6 model, the intraperitoneal tumor mass was significantly lower in the treated group compared with that of the controls (*p* = 0.05). For the BxPC3 (median survival time: control/treated = 41/65 days, *p* < 0.001) and PANC-1 (median survival time: control/treated = 19/35 days, *p* < 0.001) peritoneal dissemination models, survival analysis revealed that [^211^At]AuNP@PEG/RGD significantly prolonged overall survival. Although transient weight loss, leukopenia, and thrombocytopenia were observed at one week post-administration, a short recovery trend was evident thereafter. One month after administration, no abnormalities were found in hematological tests or histological analyses of intra-abdominal organs. **Conclusions**: The intraperitoneal administration of astatine-labeled integrin-targeted [^211^At]AuNP@PEG/RGD nanoparticles showed promising findings in terms of safety and efficacy for treating peritoneally disseminated malignant tumors.

## 1. Introduction

Despite recent advances in systemic chemotherapy, the prognosis for patients with malignancies with peritoneal dissemination remains poor [1]. In particular, pancreatic adenocarcinoma is a highly lethal disease, often with a survival of less than one year. Only 20% of patients with early-stage disease are amenable to potentially curative resection, while more than half of patients have metastatic disease [2,3]. One major metastatic pathway of pancreatic cancer is peritoneal dissemination, which is found in half of all pancreatic cancer patients at the time of death [4]. Reportedly, 9–15% of patients with advanced pancreatic cancer have microscopic peritoneal dissemination with a median survival of 6–7 weeks [5,6]. The extremely short survival in peritoneal cases likely reflects a shortened window for chemotherapy: the ascites-related gastrointestinal symptoms and reduced strength and tolerability (e.g., from hydronephrosis) limit treatment, which in turn makes second-line chemotherapy difficult to introduce [7]. In cases of peritoneal dissemination, systemic chemotherapy is usually administered in addition to cytoreductive debulking surgery [8]. However, because only a small amount of a systemically administered drug can reach the abdominal cavity, the dose acting on malignant cells seeded in the abdominal cavity is limited. Direct intraperitoneal administration of a drug can expose the seeded malignant cells to high concentrations of the drug without raising its blood concentration to toxic levels [9]; hence, intraperitoneal chemotherapy appears to be useful. Intraperitoneal chemotherapy has indeed been demonstrated to prolong life when administered as adjuvant chemotherapy [10], as has the combination of debulking surgery and intraperitoneal chemotherapy [11].

Although such treatment is in high demand, there are few suitable candidate drugs for clinical application and, to date,, there is no evidence of efficacy. Alpha-ray emitting radionuclides produce a strong cytotoxic effect by inducing DNA double-strand breaks in malignant tumors. Moreover, the radiation has a very short range and, if properly concentrated on the target, has a minimal effect on surrounding organs. If alpha-emitting radionuclides are distributed locally while suppressing their distribution to surrounding areas, the exposure to normal tissues is minimal, thus enhancing the therapeutic effect and improving safety. Astatine-211 (^211^At) emits alpha rays with a very short range (<100 μm) and a relatively short half-life (7.2 h) and can be imaged using the X-rays (77 and 79 keV) it emits.

In recent years, gold nanoparticles have attracted attention due to their wide range of potential applications, including as antimicrobial agents, anticancer drugs, and imaging agents [12]. Gold nanoparticles can be green-synthesized; their shape and size can be adjusted to suit specific applications, and their functionality can be fine-tuned, making them promising candidates for use as cores in targeted radioisotope therapeutics. We have focused on the localized retention of gold nanoparticles and their stable ability to carry astatine. To date, gold nanoparticles (AuNPs) have shown minimal diffusion when used as drug carriers in vivo, depending on the modifying molecule. We have shown that AuNPs can be stably labeled with ^211^At and that intratumoral administration of ^211^At-labeled AuNPs results in significant tissue retention and strong inhibition of tumor growth [13].

Integrins play a role in intercellular adhesion and interaction with the extracellular matrix [14]. Integrin family members are poorly expressed in normal epithelial cells [15], whereas their expression is upregulated in various malignant tumors, In particular, they serve important developmental, proliferation, and metastatic functions in tumor cells [16]. Pancreatic ductal adenocarcinoma (PDAC) accounts for 85% of pancreatic cancer diagnoses and is known to frequently metastasize to the peritoneum [17]. In PDAC, the expression of integrin α chain v is strongly associated with the proliferation of primary tumors and peritoneal dissemination [18]. The integrins containing αv that are highly expressed in PDAC are αvβ3, αvβ5, and αvβ6. It has been shown that activation of αvβ3 promotes lymph node metastasis in pancreatic cancer [19] and supports reattachment to the peritoneal epithelium and peritoneal seeding through enhanced resistance to anoikis [20]. Transcriptome analysis and protein expression analysis of multiple independent cohorts (491 cases in total) have reported that 84% to 90% of PDAC cases express integrin αvβ6 protein [21]. αvβ6 integrin overexpression is strongly associated with cancer cell invasion and metastasis and is a strong predictor of poor prognosis (hazard ratio: 2.07) [21]. Furthermore, α5 integrin is an adhesion molecule that functions as a ligand for L1CAM (CD171) and is essential for L1CAM-mediated chemotherapy resistance in these highly malignant PDAC and is associated with poor prognosis [22]. RGD peptides have been shown to be recognized by at least nine integrins (αIIbβ3, αvβ1, αvβ3, αvβ5, αvβ6, αvβ8, α3β1, α5β1, and α8β1) to date [23,24]. Therefore, RGD motifs are attractive as candidate molecules for various cancers including metastatic malignant pancreatic tumors [25]. Numerous imaging tracers and therapeutic beta-emitting radiopharmaceuticals targeting RGD have already been proposed, and their usefulness has been demonstrated [26,27,28,29]. In this study, based on the prolonged local retention of gold nanoparticles demonstrated in our previous research [13], we developed cyclic RGD-modified ^211^At-labeled gold nanoparticles as an alpha-particle-emitting agent that remains in the peritoneal cavity for an extended period after administration.

This study aimed to determine the tumor growth inhibitory effect, pharmacodynamics, and safety of ^211^At-labeled gold nanoparticles modified with RGD peptides (mPEG(Mn:350)-S-AuNP[^211^At]-*c*[RGDfK(C)] ([^211^At]AuNP@PEG/RGD); Scheme S1, Figure S1) administered intraperitoneally to an animal model of peritoneal dissemination.

## 2. Materials and Methods

### 2.1. Chemical Synthesis

Unlabeled mPEG(Mn:350)-S-AuNP-*c*[RGDfK(C)] (AuNP@PEG/RGD) and mPEG(Mn:350)-S-AuNP (AuNP@PEG) were synthesized by surface modification of AuNP with mPEG(Mn:350)-SH and *c*[RGDfK(C)] or mPEG(Mn:350)-SH (Schemes S1–S4). ^211^At was produced by ^209^Bi(α, 2n)^211^At nuclear reaction using a cyclotron and isolated using a dry distillation method. ^211^At-labeling of AuNP@PEG/RGD and AuNP@PEG were performed by shaking the reaction mixture. [^211^At]NaAt was prepared using ascorbic acid and NaHCO_3_ (Scheme S5). Details are provided in the Supplementary Materials.

### 2.2. Cell Culture

The fluorescence-expressing C6 rat glioma cell line, and human pancreatic cancer cell lines PANC-1 (RRID: CVCL_0480), MIA PaCa-2 (RRID: CVCL_0428), and BxPC3 that were either expressing or not expressing (RRID: CVCL_0186) fluorescent proteins were obtained, cultured, and prepared for transplantation purposes.

The C6 rat glioma cell line was provided by RIKEN BRC (Tsukuba, Japan) and cultured in Ham’s F-12 medium (Fujifilm Wako, Osaka, Japan) containing heat-inactivated 10% fetal bovine serum (FBS; Sigma-Aldrich, St. Louis, MO, USA) in a humidified incubator at 37 °C with 5% CO_2_. C6 glioma cells are suitable for assessing growth inhibition because they generally have a high growth rate, although seeding C6 glioma cells into the peritoneal cavity is considered an ectopic transplant. Fluorescence-expressing C6 glioma cells were established by transfecting them with a DNA plasmid encoding a red fluorescent protein (pEF1α-tdTomato Vector; Clontech, Takara Bio, Shiga, Japan). After obtaining a single clone using drug selection with G418 (Fujifilm Wako, Osaka, Japan), it was scaled up and used for experiments.

BxPC3 cells, a human pancreatic cancer cell line expressing the red fluorescence protein (RFP [DsRed2]) (#091-070, AntiCancer Japan, Chiba, Japan), were maintained in RPMI 1640 medium (Fujifilm Wako, Osaka, Japan) with 10% FBS (Corning, Corning, NY, USA) at 37 °C in a humidified atmosphere containing 5% CO_2_.

PANC-1 and MIA PaCa-2 human pancreatic cancer cells obtained from the American Type Culture Collection were cultured at 37 °C in Dulbecco’s modified Eagle medium (D-MEM) (Fujifilm Wako, Osaka, Japan) containing 10% FBS and 1% antibiotics (Fujifilm Wako, Osaka, Japan) in a humidified incubator with 5% CO_2_. Cultured cells were washed in phosphate-buffered saline (PBS) (Fujifilm Wako, Osaka, Japan) and harvested with trypsin-EDTA (Fujifilm Wako, Osaka, Japan).

### 2.3. Integrin Immunostaining for Tumors

Immunohistochemistry for integrin ανβ3 and α5β1 was performed on C6, BxPC3, PANC-1, and MIA PaCa-2 tumors grown by subcutaneous implantation in nude mice (BALB/cSlc-nu/nu) purchased from Japan SLC, Inc. (Shizuoka, Japan). The details are provided in the Supplementary Materials.

### 2.4. Cell Toxicity Study

BxPC3 cells were seeded at a density of 8000 cells/well in 1 mL of 10% FBS-supplemented high-glucose D-MEM in 24-well plates and incubated overnight. Various doses of [^211^At]AuNP@PEG/RGD were added in triplicate and incubated for 2 h at 37 °C in 5% CO_2_, and the medium was replaced with fresh medium before they were incubated for another 7 d. The medium was renewed once on day 5. Cells were then washed with PBS (500 μL/well) and fixed with 200 μL/well 4% paraformaldehyde (PFA) (Fujifilm Wako, Osaka, Japan) for 20 min at room temperature (20–25 °C). The cells in each well were washed twice with 500 μL of water, stained with 200 μL of a 0.5% crystal violet solution (20% MeOH) (Sigma-Aldrich, St. Louis, MO, USA) for 30 min, and washed with 500 μL of water again until no significant amount of crystal violet solution remained (35). For the quantification analysis, 300 μL of 0.05 M NaH2PO4 (Fujifilm Wako, Osaka, Japan) in 50% EtOH was added and 100 μL of the supernatant was transferred to a new 96-well plate and optical density at 570 nm was measured.

### 2.5. Evaluation of Intracellular Uptake of Nanoparticles

C6 rat glioma cells and BxPC3 human pancreatic cancer cells were seeded onto 35 mm glass bottom dishes (4 × 10^5^ cells/dish) in 2 mL of supplemented Ham’s F12K (Fujifilm Wako, Osaka, Japan) and D-MEM, respectively, and incubated overnight. AuNP@PEG/RGD or AuNP@PEG without RGD peptide modification were added to the dish, and the cultures were incubated for 24 h. After incubation, the cells were fixed using 4% PFA for 15 min. Then, cells were washed with PBS, stained using Hoechst 33342 trihydrochloride (Molecular Probes, Inc., Eugene, OR, USA) in the dark for 10 min at room temperature (20–25 °C), and then washed three times. The cellular uptake of nanoparticles was investigated using a Nikon A1R + inverted confocal microscope (Nikon Corp., Tokyo, Japan) using a Plan Apo VC water-immersion objective lens (60×, NA = 1.20; Nikon, Tokyo, Japan).

### 2.6. Cancer Peritoneal Dissemination Model

Animals were purchased from Japan SLC Inc. (Tokyo, Japan); they were housed in a 12 h light/12 h dark cycle and provided with food and water ad libitum. Six male nude mice (BALB/c Slc-nu/nu, 9 weeks old) were intraperitoneally implanted with 10^7^ fluorescent protein-expressing C6 cells in 200 μL of RPMI-1640 7 days before intraperitoneal administration.

Thirty male nude mice (BALB/c Slc-nu/nu, 7 weeks old) were intraperitoneally inoculated with 10^7^ RFP-expressing BxPC3 cells in 200 μL of RPMI-1640 5 d before intraperitoneal administration.

PANC-1 cells (10^7^) in 200 µL of RPMI-1640 were seeded into the abdominal cavities of 10 nude mice 5 days prior to intraperitoneal administration. The cells were suspended in the medium and intraperitoneally administered using a 26-gauge intravenous catheter.

Although C6 glioma is a cancer type that does not normally cause peritoneal dissemination, when disseminated into the peritoneal cavity of nude mice, it forms tumors at a much faster rate than pancreatic cancer cells. The purpose of establishing and testing a C6 glioma peritoneal dissemination model was to demonstrate that astatine kills tumor cells in the peritoneal cavity and inhibits tumor formation. For that purpose, this experiment was conducted as a conceptual study using a small number of animals.

### 2.7. Intraperitoneal Administration of Particles

Solutions of [^211^At]AuNP@PEG/RGD dissolved in saline, with a total volume of 1.0 mL, were prepared. [^211^At]AuNP@PEG/RGD solution or 1.0 mL saline solution was slowly infused intraperitoneally (0.979 ± 0.194 MBq for C6 models (*n* = 3), 1.139 ± 0.035 MBq for BxPC3 models (*n* = 10), and 1.308 ± 0.039 MBq for PANC-1 models (*n* = 10) per mouse) using a 26-gauge catheter. The [^211^At]AuNP@PEG solution (1.059 ± 0.075 MBq) was also administered intraperitoneally to BxPC3 model mice (*n* = 10) in the same manner. The rationale for setting the dose at approximately 1 MBq per head is that previous studies have confirmed that, when astatine is administered, safety and toxicity remain within acceptable limits as long as the dose is 1 MBq per head or less [30].

### 2.8. Evaluation of Intra-Abdominal Tumor Volume and Overall Survival

Animals implanted with C6 cells were dissected 18 d after the administration of [^211^At]AuNP@PEG/RGD or saline, and all masses were removed; animals implanted with BxPC3 cells were observed until death, which was set as the endpoint. After intraperitoneal administration, animals were observed periodically; in the C6 and BxPC3 models, body weight was measured, and whole-body imaging was performed with an in vivo compact fluorescence imaging device (ClearView™, INDEC Systems, Inc., Los Altos, CA, USA).

**Figure 1:**
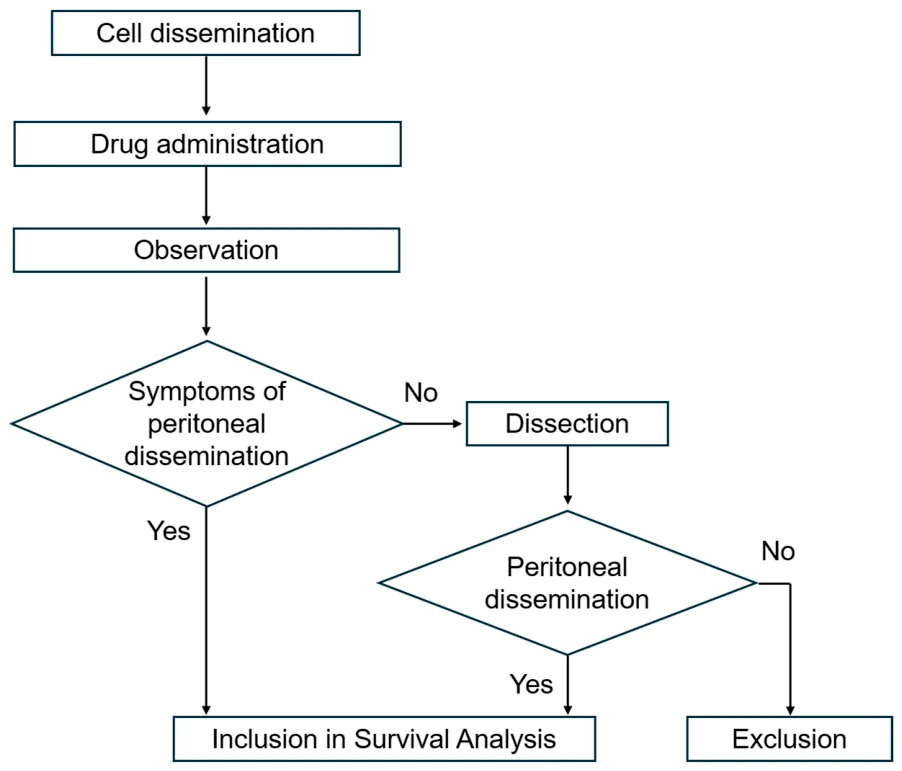
Flowchart of survival analysis for the BxPC3 and PANC-1 peritoneal dissemination models. Symptoms of peritoneal dissemination include any of the following: palpable intra-abdominal mass, accumulation of ascites, or significant weight loss (20% or more from baseline).

In the BxPC3 or PANC-1 models, if no indications of dissemination (i.e., accumulation of ascites or fluorescence in the abdomen) were observed during the observation period, dissection was performed at the time of death. The animals without peritoneal dissemination were excluded as failed models. Figure 1 shows the flowchart of the evaluation process for the BxPC3 and PANC-1 models.

### 2.9. Biodistribution Studies

For each group of two 8-week-old male ICR mice (Slc:ICR) (purchased from Japan SLC Inc., Tokyo, Japan), [^211^At]AuNP@PEG (0.423 ± 0.027 MBq/mouse) or [^211^At]AuNP@PEG/RGD (0.593 ± 0.029 MBq/mouse) was administered intraperitoneally. Mice were sacrificed and dissected 4 h and 24 h post-injection. The brain, heart, lungs, testes, bone marrow, salivary glands, and thyroid gland were harvested. During dissection, care was taken to avoid opening the abdominal cavity, and urine was collected from the bladder at the conclusion of the procedure. The extracted samples were weighed, and the radioactivity of ^211^At was measured using a gamma counter (AccuFLEX ARC γ7000, ALOKA Co., Ltd., Tokyo, Japan) on the day after administration. The measured radioactivity was decay-corrected at the time of drug administration and recorded as %ID/g. Radiation measurements were not performed on intra-abdominal organs, as intra-abdominally administered drugs adhere to organ surfaces and cause contamination.

In the case of intraperitoneal administration, since alpha-emitting radionuclides are presumed to be distributed only on the surface of the intraperitoneal organs, the radiation effects on the entire tissue are extremely limited. However, to err on the side of caution, we assumed that the radioactivity in the intra-abdominal organs was distributed uniformly throughout the tissue. We calculated the residence time using the trapezoidal approximation and then used IDAC Dose 2.1 [31] to calculate the absorbed dose for each organ.

### 2.10. Scintigraphy

Whole-body scintigraphy was performed after [^211^At]AuNP@PEG/RGD, [^211^At]AuNP@PEG, or [^211^At]NaAt administration to mice using a gamma camera (E.cam; Siemens Healthcare, Erlangen, Germany) equipped with a low-energy, high-resolution and parallel-hole collimator, according to the protocol outlined above. The detectors were set at 5 cm from the subject. Each animal was immobilized in the prone position under anesthesia with 2.0% isoflurane in oxygen, and imaging was performed for 10 min to obtain planar images of the front and rear surfaces with a 256 × 256 matrix size (pixel size: 1.2 mm).

### 2.11. Hematological and Histological Assessment

An evaluation of hematological and histological toxicity in the mice following intraperitoneal administration of [^211^At]AuNP@PEG/RGD was conducted. For comparison, the low-molecular-weight radiopharmaceutical [^211^At]NaAt was also administered intraperitoneally to assess toxicity. Details are provided in the Supplementary Materials.

### 2.12. Statistics

Statistical calculations were performed using SPSS 20 (RRID: SCR_002865, IBM Japan, Ltd., Tokyo, Japan). For two-group comparisons, the Mann–Whitney U test was used; for comparisons of three or more groups, the Kruskal–Wallis test was used. A survival function was estimated using the Kaplan–Meier method, a survival rate curve was generated, and a log-rank test was performed to compare the treated group with the control group. The *p*-value cutoff for statistical significance was set at 0.05. Multiple comparisons were adjusted using Bonferroni correction.

## 3. Results

### 3.1. AuNP Characterization

**Figure 2:**
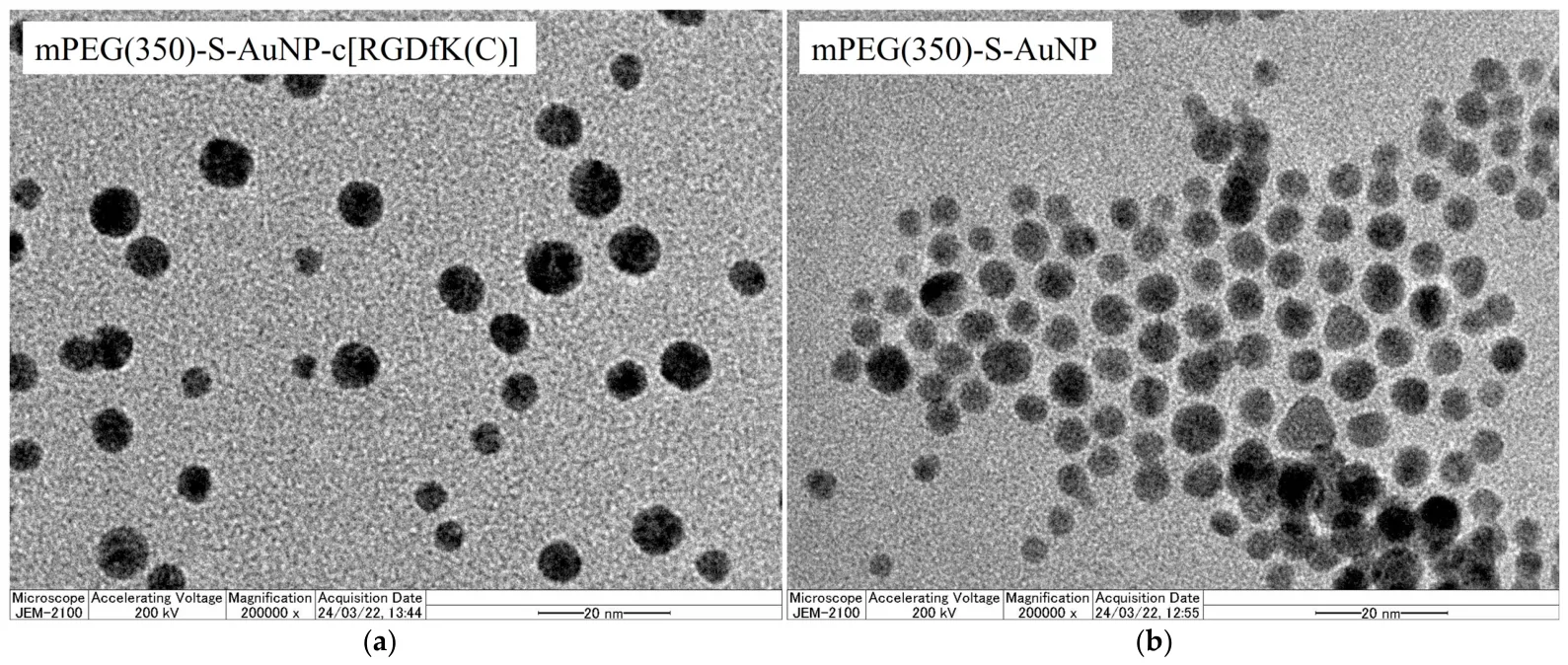
Transmission electron microscopy (TEM) images of AuNP@PEG/RGD (**a**) and AuNP@PEG (**b**) recorded using a JEM-2100 (JEOL Ltd., Tokyo, Japan).

The hydrodynamic diameter, polydispersity index, zeta-potential, and transmission electron microscopy (TEM) images of AuNP@PEG/RGD and AuNP@PEG are shown in Table 1 and Figure 2.

[^211^At]AuNP@PEG/RGD was found to adsorb to the tube after prolonged standing. It is unclear whether this adsorption is due to free At or [^211^At]AuNP@PEG/RGD. Therefore, it is difficult to evaluate the radiolabeling stability of [^211^At]AuNP@PEG/RGD.

### 3.2. In Vitro and Ex Vivo Studies

**Figure 3:**
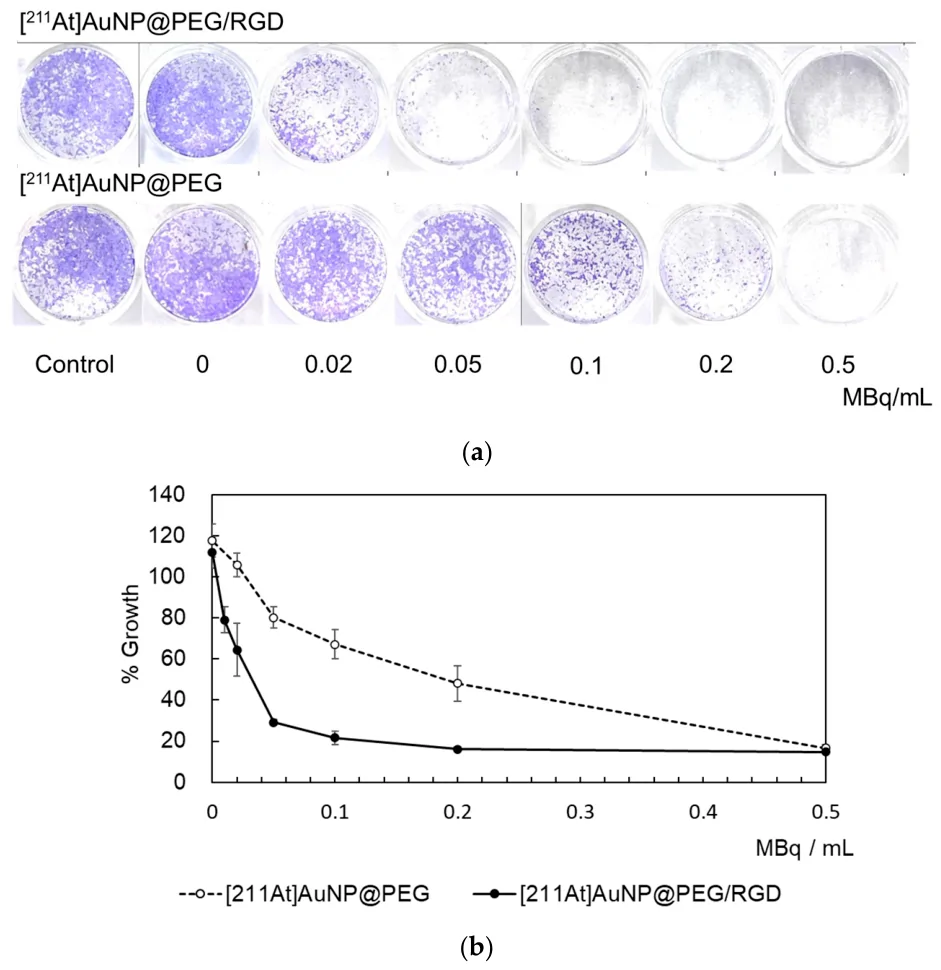
Representative images of the cell toxicity study. BxPC3 cells were treated with [^211^At]AuNP@PEG/RGD or [^211^At]AuNP@PEG at 0, 0.02, 0.05, 0.1, 0.2, and 0.5 MBq/mL of activity (**a**). The cell growth rate was evaluated by measuring the optical density at 570 nm. The number of untreated BxPC3 colonies (control) was set to 100%. Values are expressed as mean ± SD, *n* = 3 (**b**).

The immunostaining results showed high expression of α5β1 integrin in C6, BxPC3, and PANC-1 cells, and high expression of ανβ3 integrin in BxPC3 and PANC-1 cells (Figure S1). The cell toxicity study showed dose-dependent growth inhibition in BxPC3 cells treated with [^211^At]AuNP@PEG/RGD. Although [^211^At]AuNP@PEG administration showed radioactivity-dose-dependent effects, the growth rate was clearly higher than that with [^211^At]AuNP@PEG/RGD administration (Figure 3a,b).

**Figure 4:**
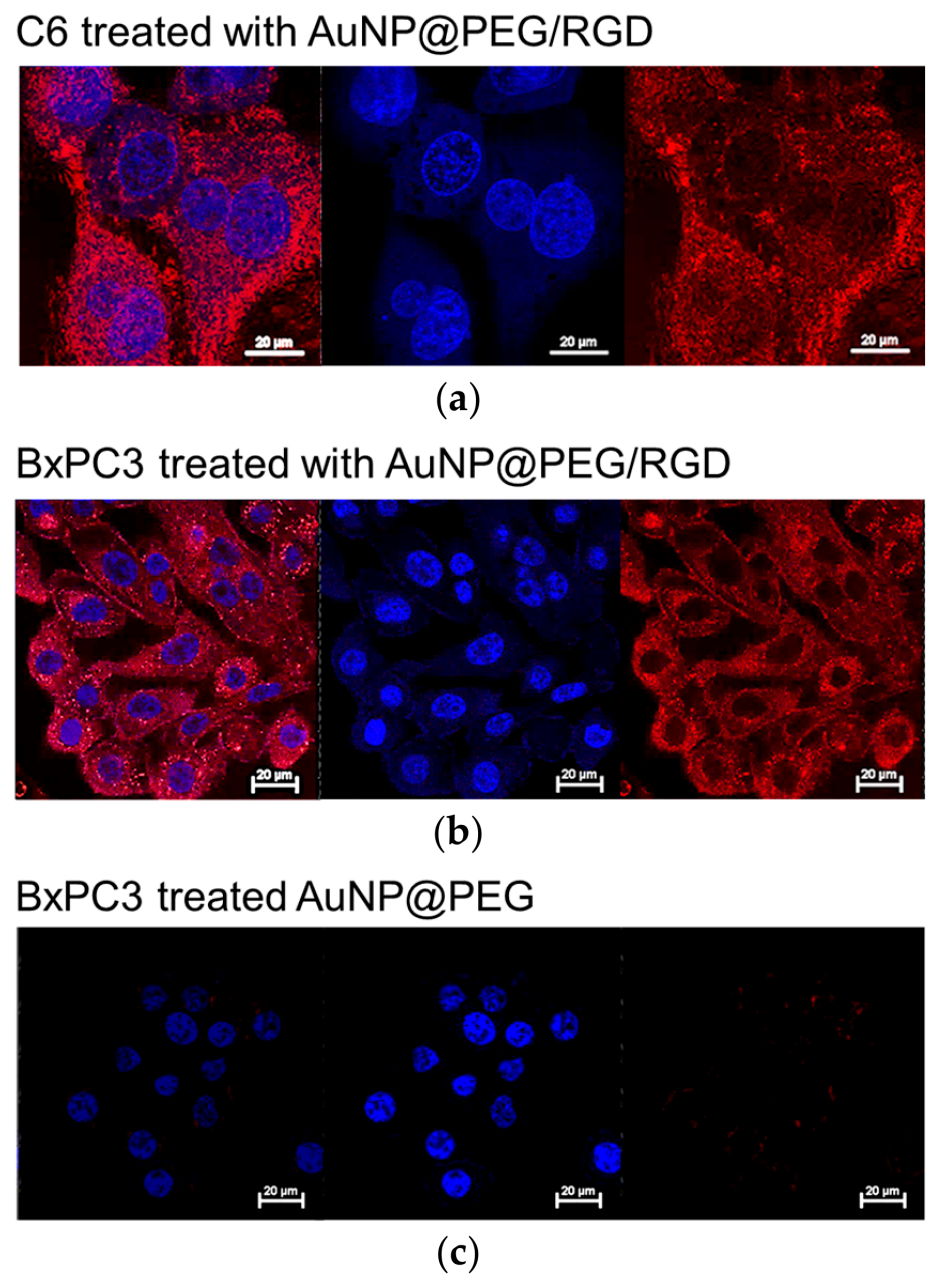
C6 glioma cells (**a**) and BxPC3 cells (**b**) were seeded onto 35 mm glass bottom dishes, and the cells were incubated with AuNP@PEG/RGD for 24 h. AuNP@PEG was administered to BxPC3 cells and cultured for 24 h (**c**). The intracellular uptake of these nanoparticles was examined using reflectance imaging. The first column shows merged images of particle reflectance (red) internalized in the cells and Hoechst-stained DNA fluorescence (blue); the second column shows Hoechst fluorescence alone, and the third column shows reflectance images of the particles taken up by the cells.

The results from the AuNP@PEG/RGD-treated C6 and BxPC3 cells confirmed that gold nanoparticles were internalized into the cells, while no clear internalization of AuNP@PEG was observed in BxPC3 cells (Figure 4).

### 3.3. Biodistribution of Administered Drugs

**Figure 5:**
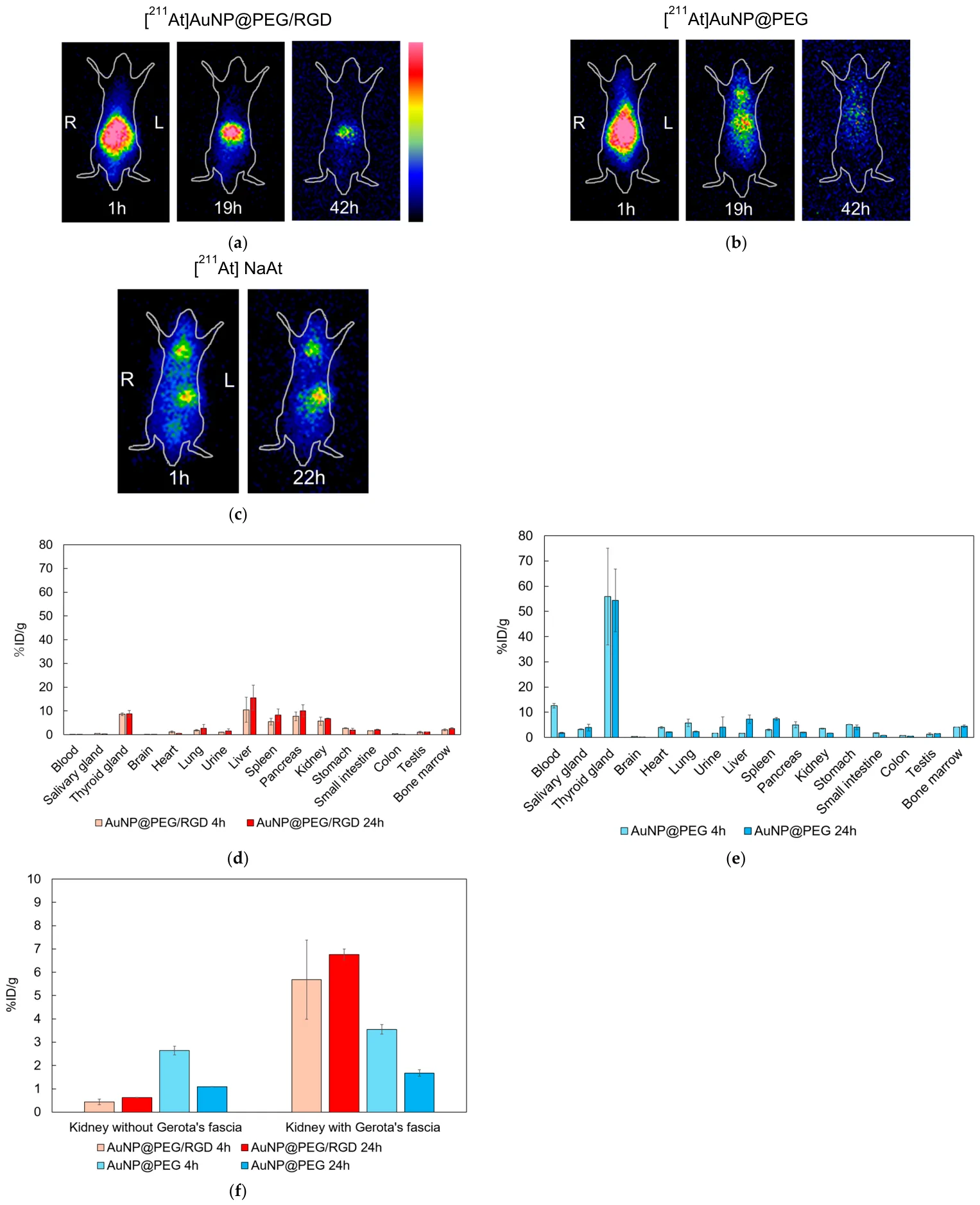
Scintigraphy of mice was performed at 1, 19, and 42 h after [^211^At]AuNP@PEG/RGD (**a**) or [^211^At]AuNP@PEG (**b**) administration, or at 1 and 22 h after [^211^At]NaAt administration (**c**). R: right, L: left. The radioactivity concentration in blood sampled from the mouse hearts was measured at 4 and 24 h after administration (**d**). Organs were removed 4 and 24 h after administration of [^211^At]AuNP@PEG/RGD (**d**) or [^211^At]AuNP@PEG (**e**), and their weights and radioactivity levels were measured. After removal, the intraperitoneal organs were washed once with PBS before radioactivity was measured. The kidneys were removed with Gerota’s fascia attached; after the initial measurement, the Gerota’s fascia was peeled away and the radioactivity was measured again (**f**).

Scintigraphy at 1, 19, and 42 h after intraperitoneal administration of [^211^At]AuNP@PEG/RGD in mice revealed that the administered radioisotope was retained in the abdominal cavity with minimal systemic diffusion (Figure 5a). In contrast, in mice administered [^211^At]AuNP@PEG, the radioisotope accumulated in the abdominal and cardiac cavity one hour after administration and subsequently diffused throughout the body (Figure 5b). In mice treated with [^211^At]NaAt, the radioisotope was rapidly distributed throughout the body, with early accumulation in the neck and left upper abdomen, which is considered to indicate thyroid and stomach uptake [30], respectively (Figure 5c).

After the administration of [^211^At]AuNP@PEG/RGD, radioactivity in the blood was extremely low, but a somewhat high accumulation was observed in the thyroid, and the radioactivity of intra-abdominal solid organs (liver, kidney, pancreas, and spleen) was somewhat high (Figure 5d). In contrast, following the administration of [^211^At]AuNP@PEG, the blood radioactivity was high, particularly in the early stages, and extremely high accumulation in the thyroid was observed over time. In addition, liver and spleen accumulation increased over time (Figure 5e). After the administration of [^211^At]AuNP@PEG/RGD, the radioactivity in the kidneys after the removal of Gerota’s fascia was markedly lower than that in the kidneys with Gerota’s fascia intact (Figure 5f). This indicates that [^211^At]AuNP@PEG/RGD remains in the peritoneal cavity and adheres at high concentrations to the organ surfaces, while internal accumulation in organs is low because the blood concentration is very low. From this, it can be inferred that for the liver, pancreas, and spleen—as with the kidney—radioactivity is likely to be distributed mainly on the organ surfaces.

Table 2 shows the absorbed doses for each major organ. When Gerota’s fascia is removed, the absorbed dose in the kidneys decreased to approximately 6% of the pre-removal value. This indicates that the absorbed doses for intra-abdominal organs were significantly overestimated.

### 3.4. Tumor Growth and Survival

**Figure 6:**
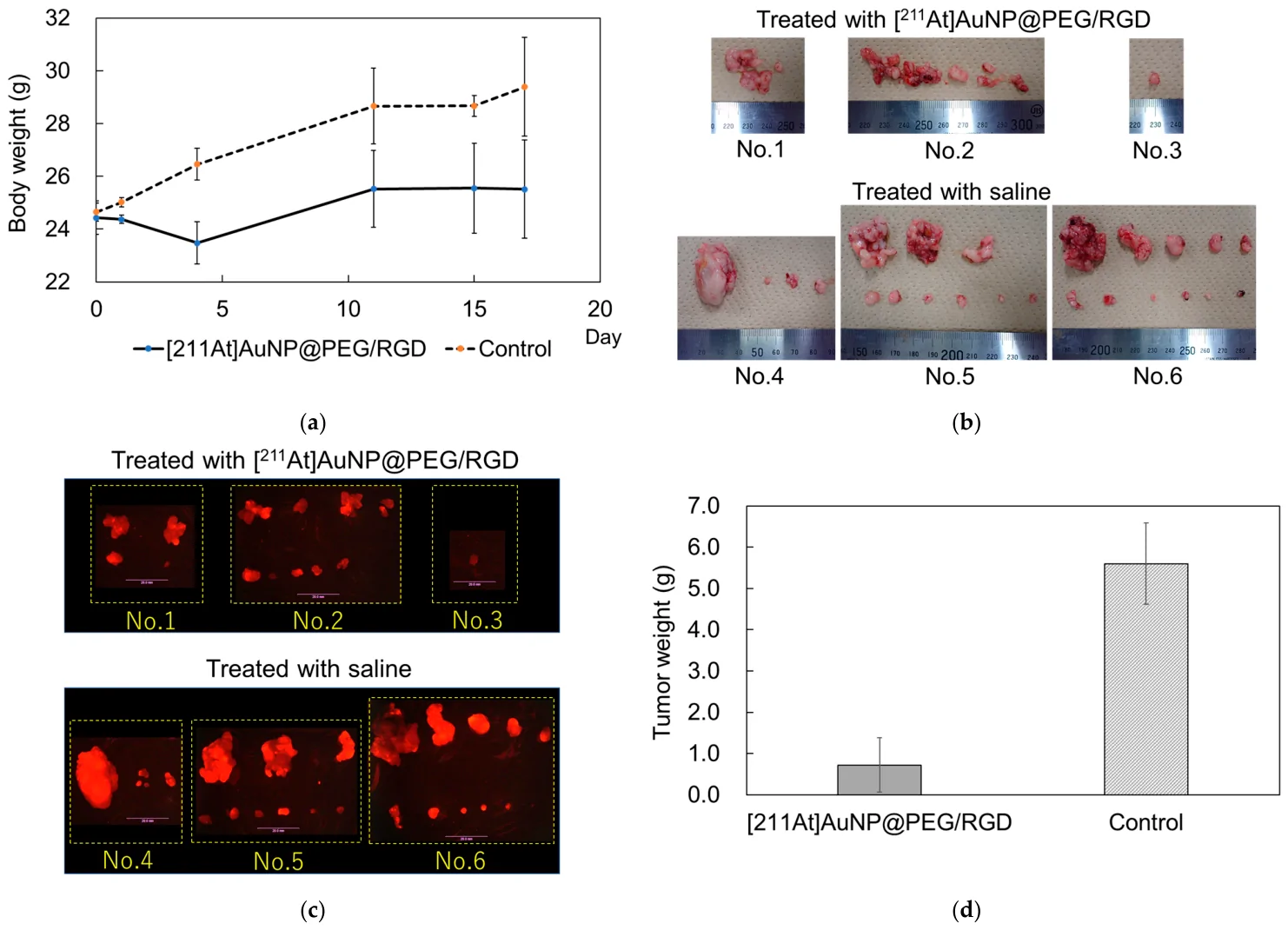
Weight changes in model and control animals are shown in (**a**). At 23 days after treatment, all tumors were collected from the abdominal cavity of the mice peritoneally seeded with C6 glioma cells (**b**), and all tumors showed fluorescence, indicating that they were C6 gliomas (**c**). Scale bars represent 20 mm. A comparison of resected tumor masses for each group is shown in (**d**).

In the C6 glioma model, control animals had large amounts of retained ascites, which caused an increase in body weight (Figure 6a). When the abdomen was opened 18 d after administration, peritoneal dissemination was observed in all animals. Fluorescence was observed in all removed masses, and tumor masses were markedly lower in animals treated with [^211^At]AuNP@PEG/RGD than in control animals (*p* = 0.05) (Figure 6b–d).

**Figure 7:**
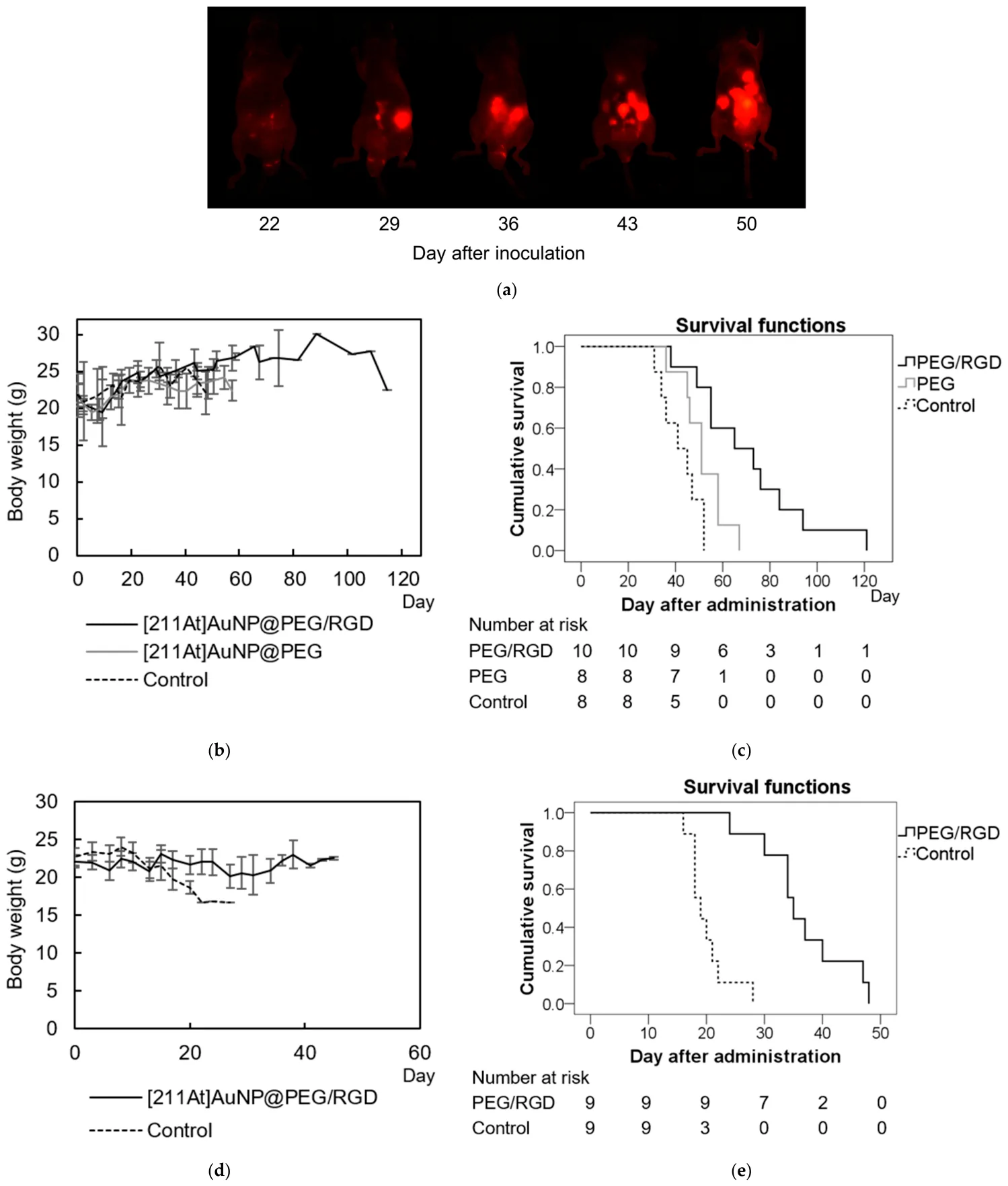
The whole bodies of nude mice with intraperitoneally disseminated fluorescent protein (RFP)-expressing BxPC3 cells were observed using a fluorescence imaging device. The representative control animal shown here (**a**) died 50 days after cell seeding. All individuals survived up to 30 days after administration. Body weight changes in BxPC3 (**b**) and PANC-1 (**d**) peritoneally seeded animal models treated with [^211^At]AuNP@PEG/RGD were compared with those of controls. In the BxPC3 and PANC-1 peritoneal seeding animal models, cumulative survival was evaluated with death as the endpoint. The BxPC3 (**c**) and PANC-1 (**e**) models treated with [^211^At]AuNP@PEG/RGD had significantly higher cumulative survival rates than the control groups. In (**c**,**e**), PEG/RGD refers to [^211^At]AuNP@PEG/RGD.

In the BxPC3 model, one of the control animals died before administration. Among the nine control mice and 10 mice treated with [^211^At]AuNP@PEG/RGD, eight and nine had findings indicating peritoneal dissemination by the time of death, respectively (Figure 7a). Although the animals treated with [^211^At]AuNP@PEG/RGD showed transient weight loss one week after administration, they recovered thereafter (Figure 7b). The overall survival of animals treated with [^211^At]AuNP@PEG/RGD was significantly longer than that of animals treated with [^211^At]AuNP@PEG (*p* = 0.01) and the control animals (*p* < 0.001) (Figure 7c) (Table 3).

In the PANC-1 model, 9/10 control mice and 9/10 mice treated with [^211^At]AuNP@PEG/RGD had findings indicating peritoneal dissemination by the time of death. Control animals showed weight loss with disease progression, whereas animals treated with [^211^At]AuNP@PEG/RGD showed early transient weight loss but no progressive weight loss (Figure 7d). Overall survival was significantly longer in animals treated with [^211^At]AuNP@PEG/RGD than in control animals (*p* < 0.001) (Figure 7e) (Table 3).

### 3.5. Hematological and Histological Adverse Effects

**Figure 8:**
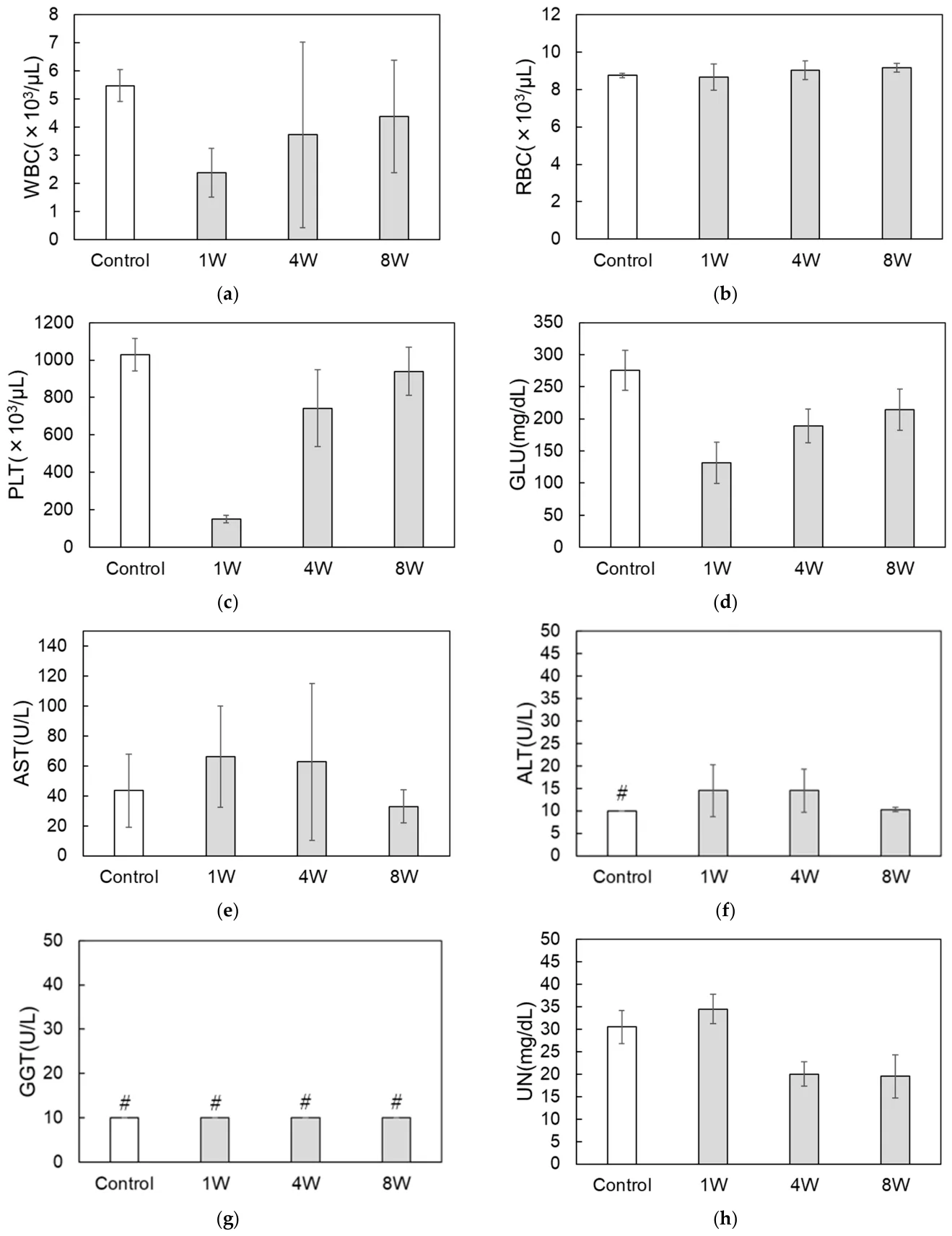

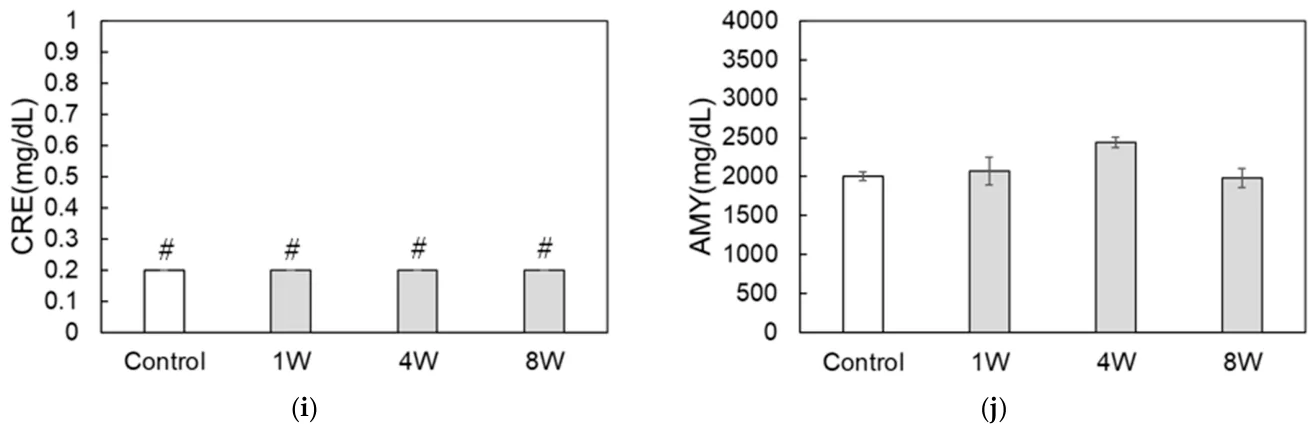
[^211^At]AuNP@PEG/RGD administration followed by blood count (**a**–**c**) and blood chemistry measurements (**d**–**j**). After blood samples were collected at each time point, the mice were euthanized (*n* = 4 at each time point). For all test items, no significant differences were detected using the Mann–Whitney U test with Bonferroni correction. WBC, white blood cell; RBC, red blood cell; PLT, platelet; GLU, glucose; AST, aspartate aminotransferase; ALT, alanine aminotransferase; GGT, gamma-glutamyltransferase; UN, urea nitrogen; CRE, creatinine; AMY, amylase; #, below the limit of quantification.

In animals treated with [^211^At]AuNP@PEG/RGD, although no abnormalities were found in the red blood cells, a transient decrease in leukocytes and platelets was observed a week after administration and recovered by 4 weeks after administration (Figure 8a–c). In animals treated with [^211^At]NaAt, although no abnormalities were found in the red blood cells, a transient decrease in platelets was observed a week after administration, and leukopenia progressed until 8 weeks after administration (Figure S2A–C). No significant abnormalities were observed in blood biochemistry tests up to 8 weeks after administration (Figure 8d–j and Figure S2D–J).

There were no apparent histological abnormalities in mesenteric, diaphragm, liver, kidney, spleen, pancreas, small intestine, or stomach tissues in normal mice treated with [^211^At]AuNP@PEG/RGD or [^211^At]NaAt at 4 weeks after administration (Figure S3A–H). In male mice treated with [^211^At]NaAt, vacuolar degeneration in the testis was observed (Figure S3I). Female mice treated with [^211^At]NaAt showed ovarian atrophy and decreased follicle development (Figure S3J). In contrast, animals treated with [^211^At]AuNP@PEG/RGD showed no evident abnormalities in their reproductive organs.

## 4. Discussion

[^211^At]AuNP@PEG/RGD was shown to be cytotoxic to malignant cells that recognize RGD peptides and to be internalized by tumor cells following exposure. When administered intraperitoneally, [^211^At]AuNP@PEG/RGD remained mostly in the peritoneal cavity until 42 h after administration. When administered intraperitoneally to the rat glioma C6 peritoneal dissemination model, it significantly inhibited intraperitoneal tumor growth compared to the controls. In the human pancreatic cancer BxPC3 or PANC-1 peritoneal dissemination model, intraperitoneal administration was found to significantly prolong overall survival. Although transient leukopenia and thrombocytopenia were noted about 1 week after [^211^At]AuNP@PEG/RGD administration, they recovered within 3 weeks. About 1 month after [^211^At]AuNP@PEG/RGD administration, there were no evident histological abnormalities in the abdominal organs.

Locoregional intraperitoneal chemotherapy can expose intraperitoneal tumor cells to high concentrations of [^211^At]AuNP@PEG/RGD for extended periods of time while maintaining low blood levels of radioactivity to reduce toxicity [4]. Intraperitoneally administered drugs have a short intraperitoneal residence time because molecules smaller than 20 kDa are rapidly removed from the abdominal cavity by direct absorption [32] and larger compounds and nanoparticles are primarily removed by lymphatic drainage through the stomata located between cuboidal mesothelial cells of the diaphragm [33]. Consequently, drug-induced side effects are relatively common, and it is difficult to increase the effectiveness of the drug by increasing the dosage. Conversely, frequent intraperitoneal administration increases the risk of problems related to inserted catheters. This necessitates reducing the frequency of drug administration, which requires sufficient drug concentrations in the abdominal cavity [34]. [^211^At]AuNP@PEG/RGD appears to be the optimal formulation for intraperitoneal administration, as scintigraphy showed that it remained in the peritoneal cavity for at least six half-lives of ^211^At. The evaluation of the extraperitoneal biodistribution also revealed that there was less [^211^At]AuNP@PEG/RGD accumulation in each organ compared to [^211^At]AuNP@PEG (Figure 5e,f). The main pathways for the peritoneal permeation of water-soluble substances are through small pores with radii of 40–55 Å or large pores with radii of approximately 250–300 Å [35,36]. Although the latter only accounts for a small proportion, and the peritoneal permeability of [^211^At]AuNP@PEG/RGD and [^211^At]AuNP@PEG is not particularly high, these particles are sorted not only by size, but also by their hydration properties and surface charge [37]. The difference in peritoneal permeability between [^211^At]AuNP@PEG/RGD and [^211^At]AuNP@PEG may be due to the difference in zeta potential or nanoparticle clustering within the peritoneal cavity.

When [^211^At]AuNP@PEG/RGD is administered intraperitoneally, astatine accumulates in the thyroid at a concentration of 10% ID/g or less (Figure 5d). Although this accumulation is significantly lower than that of [^211^At]AuNP@PEG, it may pose a problem at clinical doses. For clinical applications, it is considered preferable to administer iodine prior to drug administration [38].

C6, BxPC3, and PANC-1 cells are all known to express integrins that recognize RGD motifs [15,23,39,40]. In our study, BxPC3 cells showed particularly strong expression αvβ3, and C6 and PANC-1 cells strongly expressed α5β1 (Figure S1). After treatment with [^211^At]AuNP@PEG/RGD, cytotoxicity was observed in BxPC3, while no or weak cytotoxicity was observed when [^211^At]AuNP@PEG without cyclic RGD peptide modification was administered (Figure 3). Thus, cellular injury appears to be mediated by binding of the cyclic RGD peptide to integrins expressed on cells. In addition, the cytotoxicity was dependent on administered radioactivity, which is an effect of alpha radiation.

[^211^At]AuNP@PEG/RGD was found to internalize into C6 and BxPC3 cells after administration in the presence of cyclic RGD (Figure 4). Because of the short range of ^211^At alpha rays, the source must be internalized into the cell to effectively damage DNA. Cellular internalization, as observed here, would bring ^211^At into proximity to the nucleus, resulting in DNA double-strand breaks. In most cases, small particles such as [^211^At]AuNPs, which enter cells via endocytosis, may overcome or reduce drug resistance due to overexpression of drug efflux pumps [41]. This feature of [^211^At]AuNPs is favorable for alpha irradiation with ^211^At, which has a half-life of 7 h, thus minimizing radiation effects on other organs.

Various anticancer agents that use RGD as a targeting molecule have already been developed, including RGD-modified nanoparticles [42]. In particular, Dijkgraaf et al. reported the pharmacokinetic and antitumor effects of intraperitoneal administration of ^177^Lu-DOTA-cRGDfK as a radioisotope therapeutic agent for peritoneal dissemination in a model peritoneally seeded with human ovarian cancer OVCAR-3 cells [43]. In their study, the survival rate of mice treated intraperitoneally with ^177^Lu-DOTA-cRGDfK was significantly increased compared to untreated controls. However, their research did not adequately addressed safety and toxicity concerns. Furthermore, they reported that the degree of accumulation in systemic organs did not differ clearly between the two groups when the drug was administered intraperitoneally compared to intravenous administration. In our study, when [^211^At]NaAt was administered intraperitoneally, the radioisotope diffused extraperitoneally, resulting in more prolonged thrombocytopenia and greater effects on radiosensitive reproductive organs compared to [^211^At]AuNP@PEG/RGD. Systemic diffusion of radionuclides with significant biological effects must be kept as low as possible. Furthermore, when seeking pharmacological effects within the peritoneal cavity, it is most important that the drug remain within the peritoneal cavity rather than being distributed systemically [44].

This study has several limitations. It was unable to provide any direct evidence of the binding of the targeted molecule (*c*RGD) to malignant cells within the peritoneal cavity. However, attempting to verify this in vivo using an integrin inhibitor raises the problem that the results would be confounded by the antitumor effects of the inhibitor. In this study, as indirect evidence of the efficacy of the targeted molecule, we evaluated the overall survival rate of the treatment group administered [^211^At]AuNP@PEG/RGD, using animals administered [^211^At]AuNP@PEG without *c*RGD modification as a control group. Since this study is a pilot study, only a single-dose trial was conducted in vivo. In vitro, the efficacy was shown to be dose-dependent, and given the drug’s low toxicity, we believe that further high-dose trials are necessary during the drug discovery phase. In this study, when evaluating the distribution of radioactivity in vivo, the absorbed dose was calculated based on the assumption that radioactivity was uniformly distributed throughout the entire organ, even though it was actually adsorbed on the surface of the intraperitoneal organs. In the case of alpha-emitting radiopharmaceuticals, this method results in a significant overestimation. It is necessary to establish a highly accurate method for evaluating the absorbed dose based on organ autoradiography. This study evaluated hematologic toxicity for up to 2 months after administration and histological effects for up to 1 month after administration. We believe that, at the stage of clinical application, longer-term safety evaluations are necessary to assess delayed radiation effects.

## 5. Conclusions

[^211^At]AuNP@PEG/RGD showed minimal blood transfer after intraperitoneal administration and remained in the abdominal cavity for up to 42 h. Intraperitoneal administration of [^211^At]AuNP@PEG/RGD to mice seeded intraperitoneally with malignant tumor cells expressing RGD peptide-recognizing integrins resulted in significant therapeutic effects. No evident histological abnormalities in intraperitoneal organs were observed, although transient hematopoietic disturbances were observed after intraperitoneal administration. [^211^At]AuNP@PEG/RGD is a promising therapeutic candidate for treating peritoneal dissemination of malignant tumors that recognize the RGD motif.

## Figures and Tables

**Table 1:** Hydrodynamic diameter, polydispersity index, and zeta-potential of AuNP@PEG/RGD and AuNP@PEG.

	Hydrodynamic Diameter (nm)	Polydispersity Index	Zeta-Potential
AuNP@PEG/RGD	14.37 ± 0.9252	0.1890	−8.534 ± 1.679
AuNP@PEG	13.54 ± 0.2292	0.1829	−0.8286 ± 0.2148

Measured using a Zetasizer Ultra (Malvern Panalytical). Samples were measured in 10 mM HEPES buffer (*n* = 3).

**Table 2:** Estimated absorbed dose in different tissues.

Organ/Tissue	Absorbed Dose (mGy/MBq)
Brain	1.1 × 10^−3^
Colon wall	1.0 × 10^−1^
Heart wall	4.6 × 10^−2^
Kidneys	9.0 × 10^−1^
Kidneys without Gerota’s fascia	5.5 × 10^−2^
Liver	2.2
Lung	9.9 × 10^−2^
Pancreas	1.5
Red (active) bone marrow	5.1 × 10^−2^
Salivary glands	1.5 × 10^−1^
Small intestine wall	6.1 × 10^−1^
Spleen	5.1 × 10^−1^
Stomach wall	2.2
Testes	1.1
Thyroid	8.7 × 10^−1^
Urinary bladder wall	1.6 × 10^−1^

**Table 3:** Results of survival analysis.

Tumor	Drug	Median	Confidence Interval	*p* Value vs. Control	*p* Value vs. [^211^At]AuNP@PEG
BxPC3	Saline (control)	41	28.5–53.5	-	N.S.
	[^211^At]AuNP@PEG	51	44.3–57.7	N.S.	-
	[^211^At]AuNP@PEG/RGD	65	37.1–92.9	<0.001	0.025
PANC-1	Saline (control)	19	16.1–21.9	-	-
	[^211^At]AuNP@PEG/RGD	35	32.1–37.9	<0.001	-

N.S.: not significant.

## Data Availability

The data presented in this study are available upon request from the corresponding author.

## Supplementary Materials

The following supporting information can be downloaded at: https://www.mdpi.com/article/10.3390/pharmaceutics18091102/s1, Scheme S1: Synthesis of mPEG(Mn:350)-S-AuNP-c[RGDfK(C)]; Scheme S2. Synthesis of mPEG(Mn:350)-S-AuNP; Scheme S3: Synthesis of [^211^At]AuNP@PEG/RGD; Scheme S4. Synthesis of [^211^At]AuNP@PEG; Scheme S5: Synthesis of [^211^At]NaAt; Figure S1: Integrin immunohistological staining of tumor cells. The nucleus (DAPI: blue) and integrin (antibody: red) are shown as fused images. Immunohistochemistry of integrin ανβ3 was performed on C6 (A), BxPC3 (B), PANC-1 (C), and MIA PaCa-2 (D). Clear expression of integrin ανβ3 was observed in BxPC3 and PANC-1, while expression was weak in C6 and MIA PaCa-2. Immunohistochemistry for integrin α5β1 was also performed on C6 (E), BxPC3 (F), PANC-1 (G), and MIA PaCa-2 (H). Integrin α5β1 expression was observed in C6, BxPC3, and PANC-1, showing particularly strong expression in C6 and PANC-1; Figure S2: Hematological evaluation after [^211^At]NaAt administration. Blood counts (white and red blood cell and platelet counts) (A–C) and blood chemistry tests (D–J) were performed using the above samples and evaluated based on the Kruskal–Wallis test with Bonferroni correction. Although no statistically significant difference was observed, one week after treatment with [^211^At]AuNP@PEG/RGD, platelet counts decreased compared to the controls. WBC, white blood cell; RBC, red blood cell; PLT, platelet; GLU, glucose; AST, aspartate aminotransferase; ALT, alanine aminotransferase; GGT, gamma-glutamyltransferase; UN, blood urea nitrogen; CRE, creatinine; AMY, amylase. #: Below the limit of quantification; Figure S3: Histological evaluation of intra-abdominal organs after treatment. One week after saline administration or four weeks after [^211^At]AuNP@PEG/RGD or [^211^At]NaAt administration, the mesentery (A), diaphragm (B), liver (C), kidney (D), spleen (E), pancreas (F), stomach (G), small intestine (H), testis (I), and ovary (J) were harvested and histologically evaluated by HE staining. Leftmost, middle, and rightmost, organ specimens of mice indicate saline, [^211^At]NaAt, and [^211^At]AuNP@PEG/RGD treatment, respectively. NaAt: [^211^At]NaAt, AuNP: [^211^At]AuNP@PEG/RGD.

## Abbreviations

The following abbreviations are used in this manuscript:

AuNP	Gold nanoparticle
PDAC	Pancreatic ductal adenocarcinoma
L1CAM	L1 Cell Adhesion Molecule
RGD	Arginine (Arg), Glycine (Gly), and Aspartic acid (Asp)
PEG	Polyethylene Glycol
Mn	Number-Average Molecular Weight
RFP	Red fluorescent protein
ARRIVE	Animal Research: Reporting of In Vivo Experiments
WBC	White blood cell
RBC	Red blood cell
PLT	Platelet
GLU	Glucose
AST	Aspartate aminotransferase
ALT	Alanine aminotransferase
GGT	Gamma-Glutamyltransferase
UN	Urea nitrogen
CRE	Creatinine
AMY	Amylase
DOTA	Dodecane Tetraacetic Acid
