# Supplementary material for: Therapeutic Efficacy and Safety of Intraperitoneally Administered 211At-Labeled Gold Nanoparticles for Peritoneally Disseminated Malignancies
Source: Pharmaceutics. 2026 Sep 2;18(9):1102. doi: 10.3390/pharmaceutics18091102 (PMC13610389; doi:10.3390/pharmaceutics18091102)
Supplement: Supplementary file 1 [file pharmaceutics-18-01102-s001.zip › pharmaceutics-4516242-supplementary.pdf]

Supplemental Data

AuNP modification and characterization

Scheme S1. Synthesis of mPEG(Mn:350)-S- AuNP-*c*[RGDfK(C)]

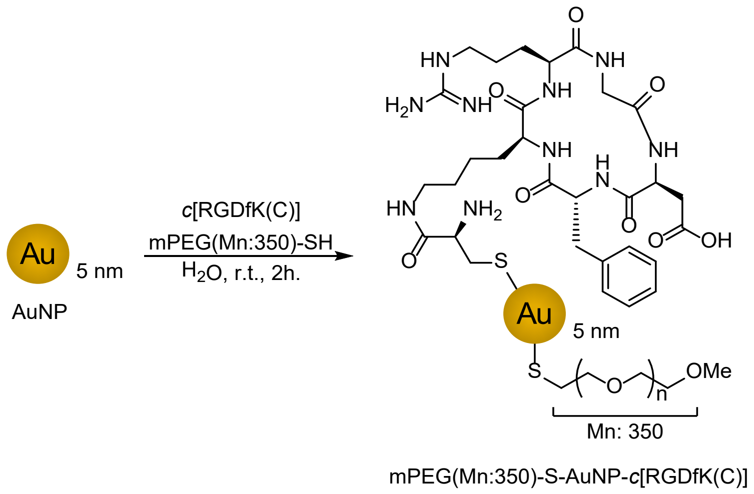

mPEG(Mn:350)-S-AuNP-*c*[RGDfK(C)] (AuNP@PEG/RGD) modification was achieved by adding *c*[RGDfK(C)]/mPEG(Mn:350)-SH (1:1.4) to 5 nm AuNP solution with a final concentration of 0.07 mM (*c*[RGDfK(C)]) and 0.1 mM (mPEG(Mn:350)-SH) and mixing for 2 h at room temperature. Subsequently, it was purified using a 50k filter device (Amicon Ultra) by centrifugation (10,000 × *g* for 5 min at 4 °C) and finally diluted in distilled water.

Scheme S2. Synthesis of mPEG(Mn:350)-S-AuNP

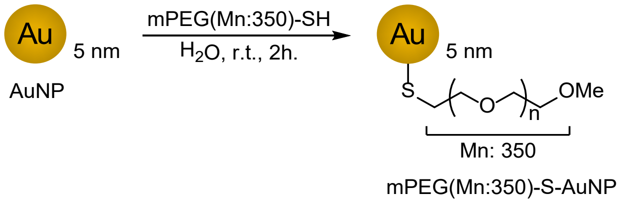

mPEG(Mn:350)-S-AuNP (AuNP@PEG) modification was achieved by adding mPEG(Mn:350)-SH to 5 nm AuNP solution with a final concentration of 0.1 mM and mixing for 2 h at room temperature. Subsequently, it was purified using a 50k filter device (Amicon Ultra) by centrifugation (10,000 × g for 5 min at 4 °C) and finally diluted in distilled water.

^{211}At-labeling of surface-modified AuNP(s)

Scheme S3. Synthesis of [^{211}At]AuNP@PEG/RGD

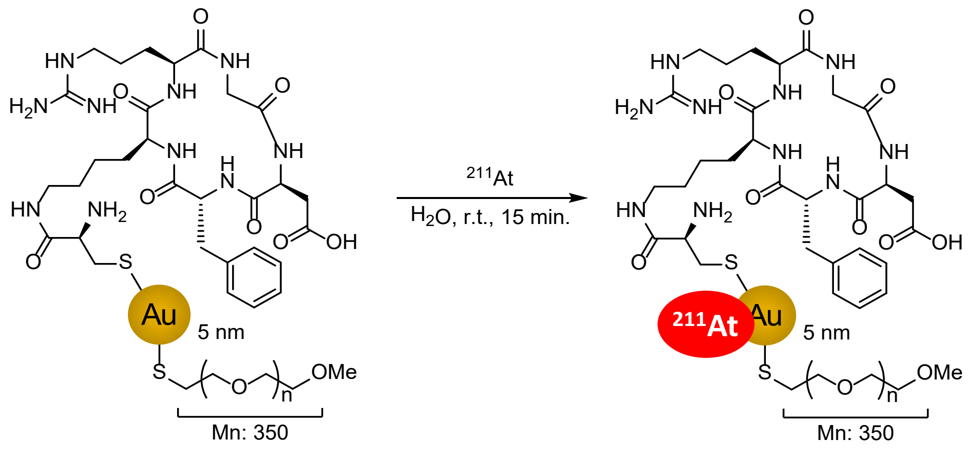

mPEG(Mn:350)-S-AuNP-*c*[RGDfK(C)]   mPEG(Mn:350)-S-AuNP[^{211}At]-*c*[RGDfK(C)]

^{211}At in H_2O was added to a suspension of AuNP@PEG/RGD in H_2O at room temperature (20 - 25°C) and the reaction mixture was shaken at room temperature for 15 min. After shaking, [^{211}At]AuNP@PEG/RGD (c.a. 63 MBq/mL) was obtained. For the animal experiments, [^{211}At]AuNP@PEG/RGD was directly used without purification. For colony-assay experiments, [^{211}At]AuNP@PEG/RGD was purified by ultrafiltration using a 50k filter device (Amicon Ultra).

Scheme S4. Synthesis of [^{211}At]AuNP@PEG

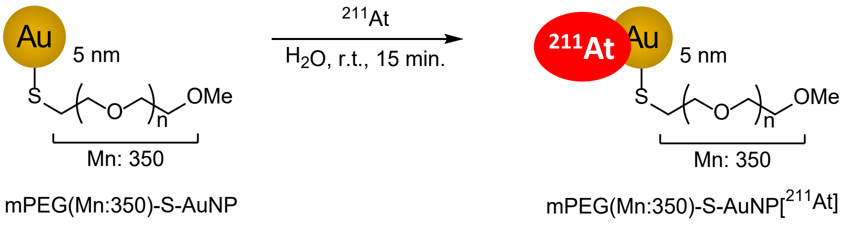

^{211}At was added to a suspension of AuNP@PEG in H_2O at room temperature, and the reaction mixture was shaken at room temperature for 15 min. After shaking, [^{211}At]AuNP@PEG (c.a. 69 MBq/mL) was obtained. For colony-assay experiments, [^{211}At]AuNP@PEG was purified by ultrafiltration with a 50k filter device (Amicon Ultra).

Synthesis of [^{211}At]NaAt

Scheme S5. Synthesis of [^{211}At]NaAt.

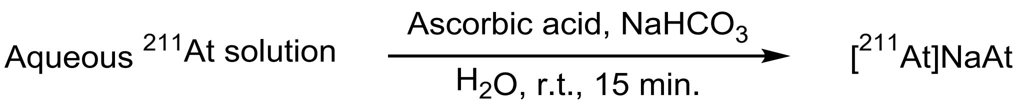

Aqueous ^{211}At solution (20.5 μL) was added to a solution of ascorbic acid (L-ascorbic acid, A0537, Tokyo Chemical Industry Co., Ltd., Japan) and $NaHCO_3$ in H_2O (100 μL) was added to at room temperature, and the reaction mixture was shaken at room temperature for 15 min. After shaking, [^{211}At]NaAt (4.33 MBq) was obtained. After

another 30 min, [^{211}At]NaAt was used for animal experiments.

Integrin immunostaining for tumors

C6 glioma cells (without fluorescent tag) (1.0 × 10^7 cells/50 µL MEM), human pancreatic cancer PANC-1, BxPC3 (without fluorescent tag), or MIA PaCa-2 cells (1.0 × 10^7 cells/50 µL RPMI1640), supplemented with 50 µL matrigel (Corning, New York, USA), were subcutaneously transplanted into the right flank of 7-week-old male nude mice (BALB/cSlc-nu/nu) purchased from Japan SLC, Inc., (Shizuoka, Japan), under anesthesia with 2.0% isoflurane in oxygen. One month after implantation for PANC-1 or MIA PaCa-2 and two months after implantation for BxPC3, subcutaneous tumors were harvested, embedded in OCT compound, and frozen at -80 °C to make frozen blocks, which were sliced thinly (5 µm) in a cryostat. After air-drying for at least 1 h, tissue sections were washed thrice over 5 min with 0.01 M PBS. For immunostaining, the tissue sections were incubated in PBS containing 0.3% Triton-X (PBS-T) (Sigma-Aldrich, St. Louis, Missouri, USA) and treated with 1% bovine serum albumin (BSA) (Sigma-Aldrich, St. Louis, Missouri, USA) for 60 min at room temperature. Tumor sections were stained with primary antibody solution containing phycoerythrin conjugated anti-integrin αvβ3, clone LM609 (1:100; MAB1976Z, Millipore, RRID: AB_11212117, Merck Life Science,

Japan), or anti-Integrin α5β1 antibody, clone BMB5 (1:100; MAB2514; Millipore, RRID: AB_94626, Merck Life Science, Japan), and nuclei were stained using 4′ ,6-diamidino-2-phenylindole (DAPI) (1:1000; D523; Dojindo, Japan) overnight at room temperature. Stained sections were then washed twice with wash buffer (DPBS + 0.5% BSA) and stained with dye-conjugated secondary antibody solution containing the donkey antirat IgG H&L AlexaFluor 568 preadsorbed (1:100; ab1754751; Abcam Plc, Cambridge, UK) and 0.3% Triton X-100 in 0.01 M PBS at room temperature for 1 h. After washing thrice over 5 min in 0.01 M PBS, tissue sections were mounted with Fluoromount™ (Diagnostic BioSystems, USA). Cells were visualized by a fluorescent microscopy (BZ9000, KEYENCE Corporation, Japan).

Figure S1. Integrin immunohistological staining of tumor cells

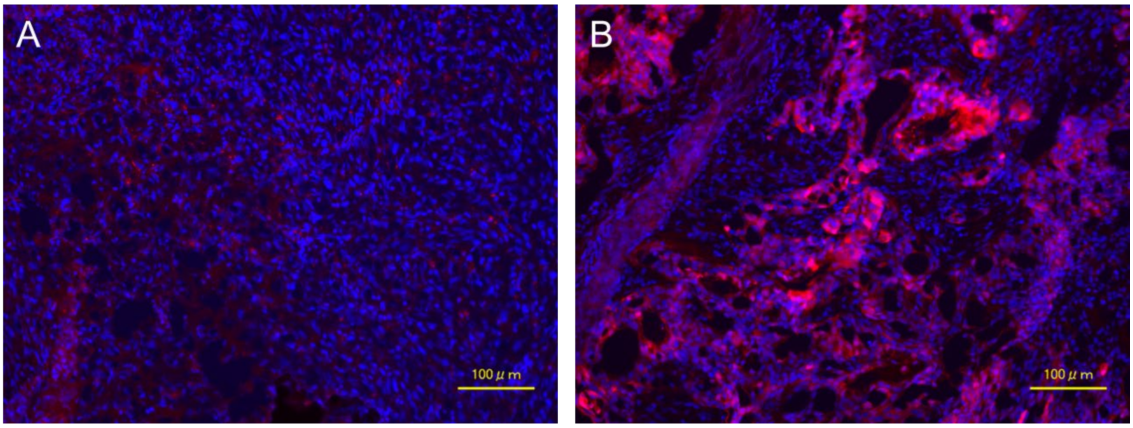

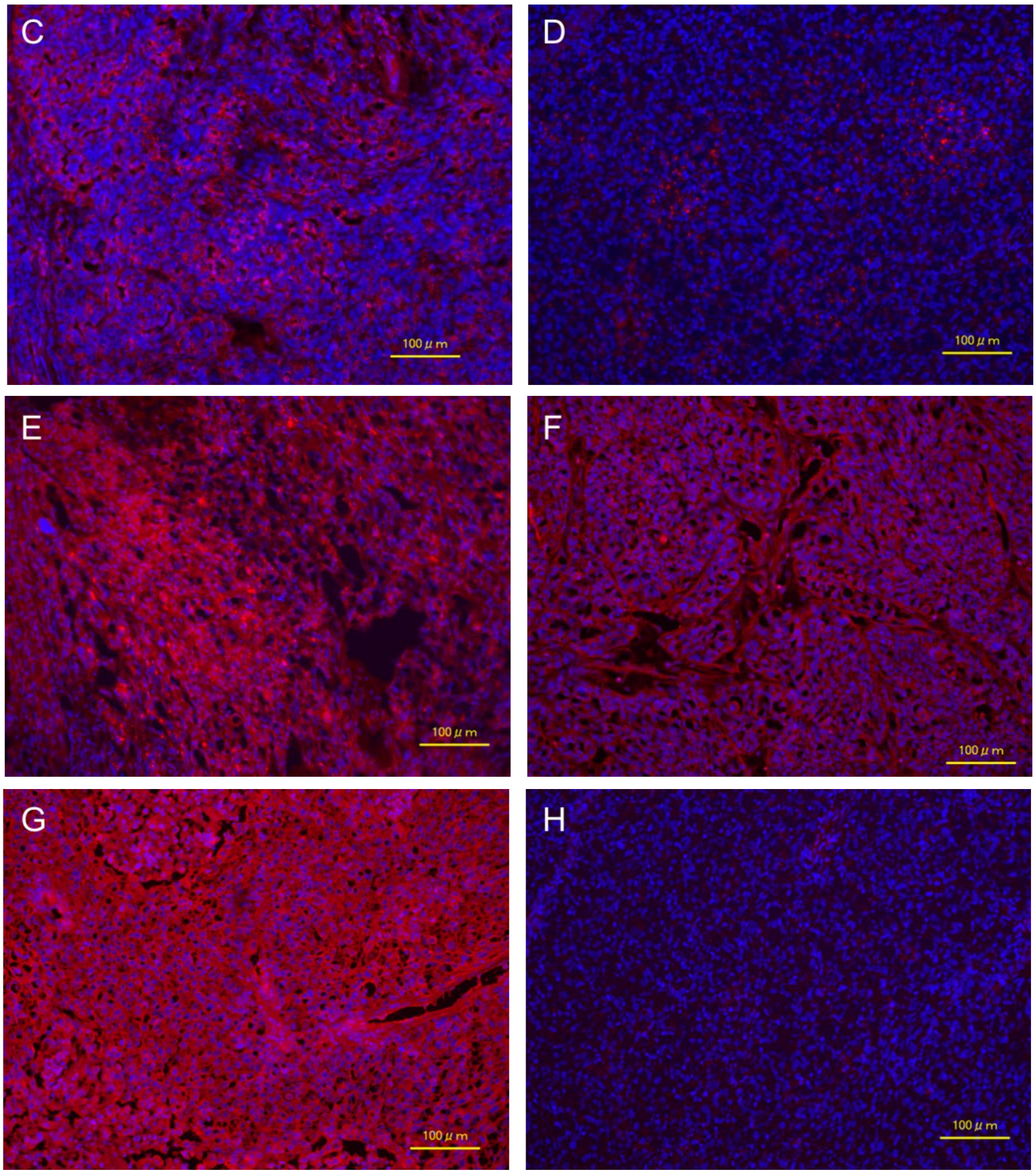

The nucleus (DAPI: blue) and integrin (antibody: red) are shown as fused images. Immunohistochemistry of integrin αvβ3 was performed on C6 (A), BxPC3 (B), PANC-1 (C), and MIA PaCa-2 (D). Clear expression of integrin αvβ3 was observed in BxPC3 and PANC-1, while expression was weak in C6 and MIA PaCa-2. Immunohistochemistry for

integrin α5β1 was also performed on C6 (E), BxPC3 (F), PANC-1 (G), and MIA PaCa-2 (H). Integrin α5β1 expression was observed in C6, BxPC3, and PANC-1, showing particularly strong expression in C6 and PANC-1.

Hematological and histological assessment

[^{211}At]AuNP@PEG/RGD was diluted in saline to 0.5 mL, and each was administered intraperitoneally to 8-week-old male nude mice (1.080 ± 0.130 MBq) and 8-week-old female nude mice (0.935 ± 0.174 MBq). After 7 d (n = 4), 4 weeks (n = 3), and 8 weeks (n = 4) of treatment, each nude mouse was sacrificed by drawing blood from its heart. Blood counts and blood biochemistry tests were performed on the blood samples for male animals treated with [^{211}At]AuNP@PEG/RGD. In addition, blood was also collected from the cardiac puncture of control mice 1 week after intraperitoneal saline administration. After 4 weeks of treatment with [^{211}At]AuNP@PEG/RGD mesenteric, diaphragm, liver, kidney, spleen, pancreas, small intestine, stomach, and testis/ovary (male [n = 3]; female [n = 2]) were extracted from the mice. The excised tumor tissues were thinly sliced into 5 µm slices and embedded in paraffin blocks, and hematoxylin–eosin staining was performed.

To compare the hematological and histological effects with those of systemic astatine distribution, [^{211}At]NaAt was administered intraperitoneally to male (1.229 ± 0.024 MBq)

and female (0.886 ± 0.131 MBq) mice, and the same evaluations as above were performed.

Figure S2. Hematological evaluation after [^{211}At]NaAt administration

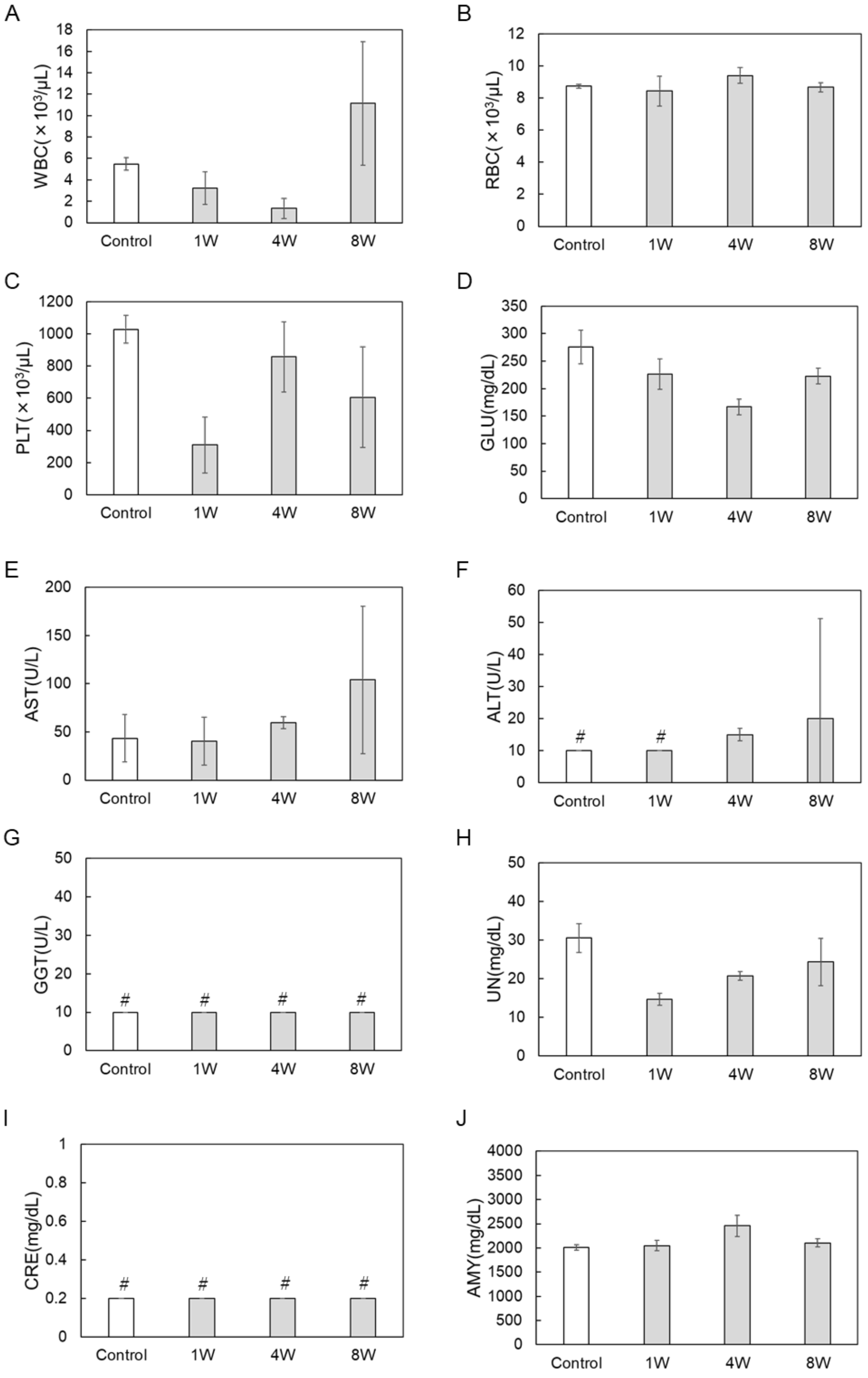

Blood counts (white and red blood cell and platelet counts) (A – C) and blood chemistry tests (D – J) were performed using the above samples and evaluated based on the Kruskal–Wallis test with Bonferroni correction. Although no statistically significant difference was observed, one week after treatment with [^{211}At]AuNP@PEG/RGD, platelet counts decreased compared to the controls.

WBC, white blood cell; RBC, red blood cell; PLT, platelet; GLU, glucose; AST, aspartate aminotransferase; ALT, alanine aminotransferase; GGT, gamma-glutamyltransferase; UN, blood urea nitrogen; CRE, creatinine; AMY, amylase.

#, Below the limit of quantification.

Figure S3. Histological evaluation of intra-abdominal organs after treatment

A

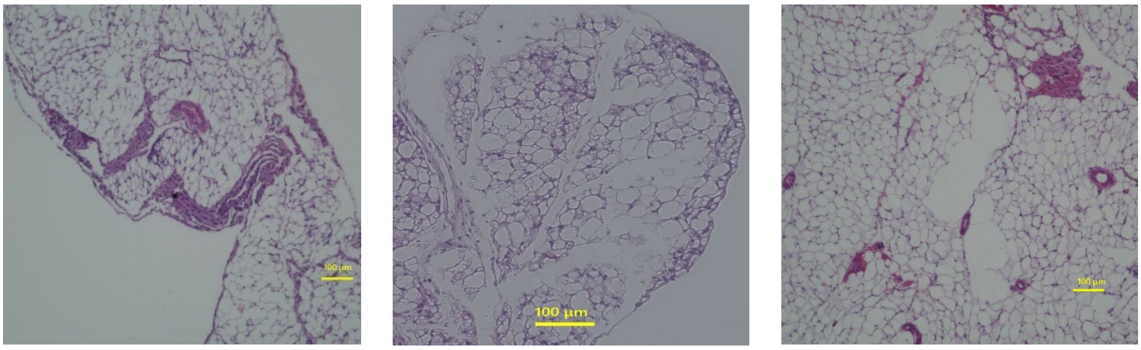

B

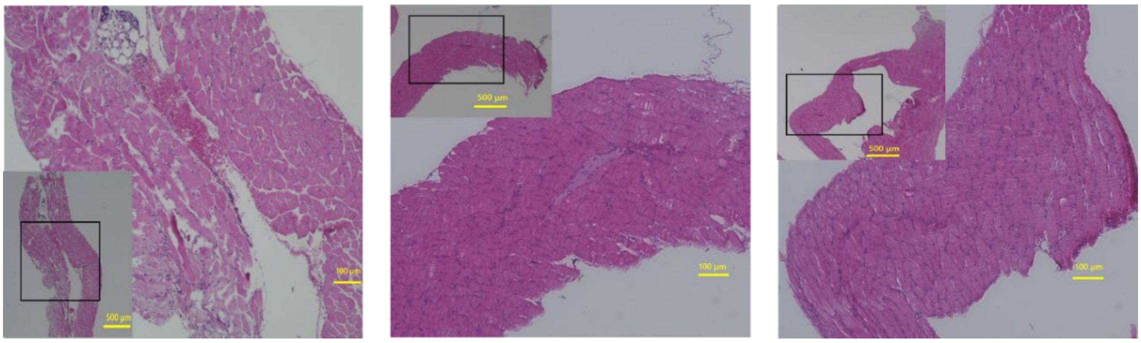

C

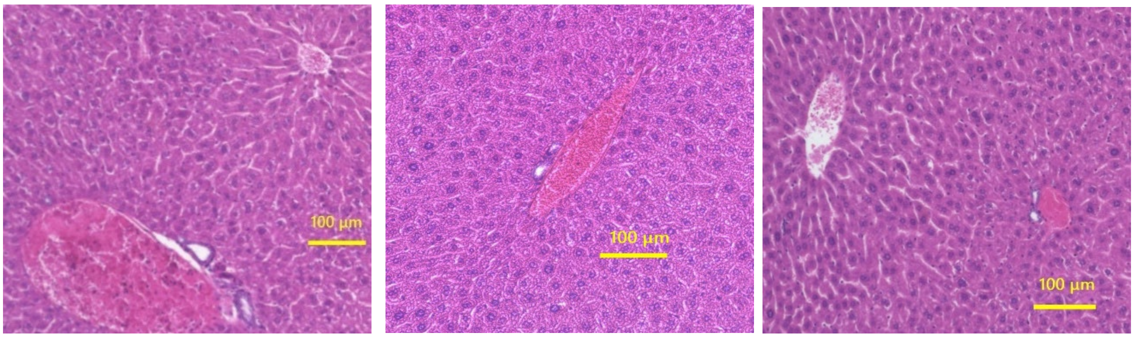

D

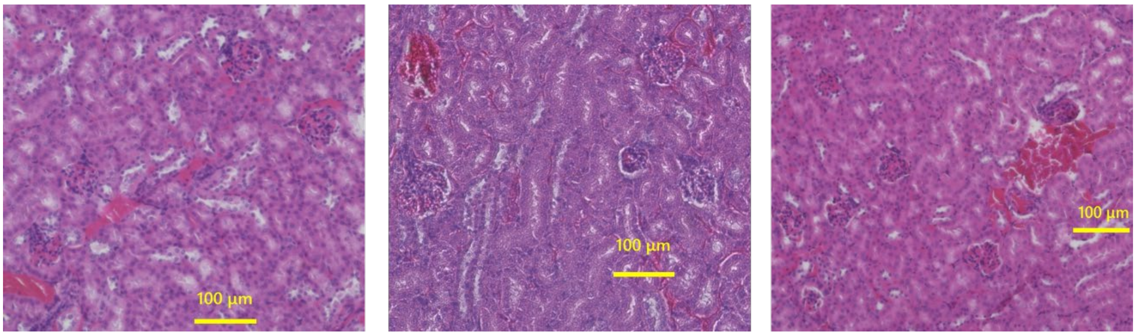
100 µm
100 µm
100 µm

E

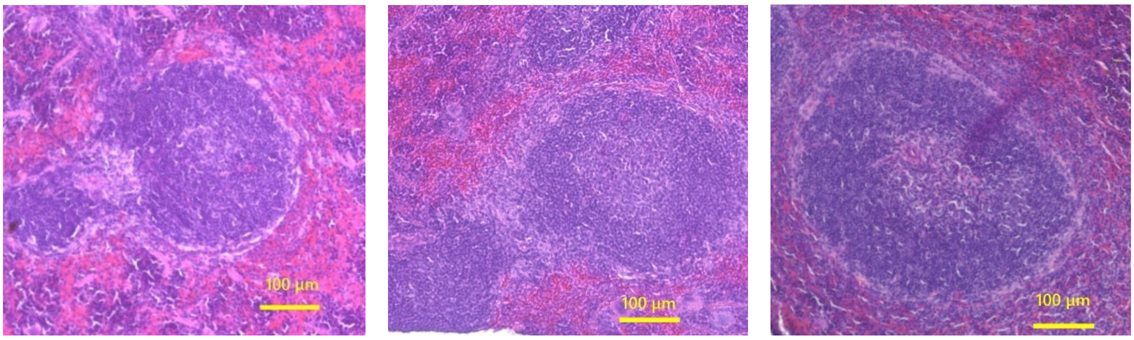
100 µm
100 µm
100 µm

F

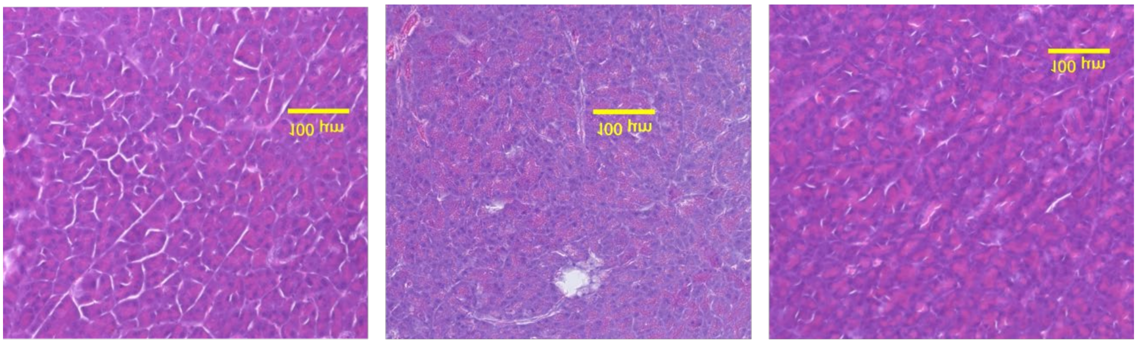
100 µm
100 µm
100 µm

G

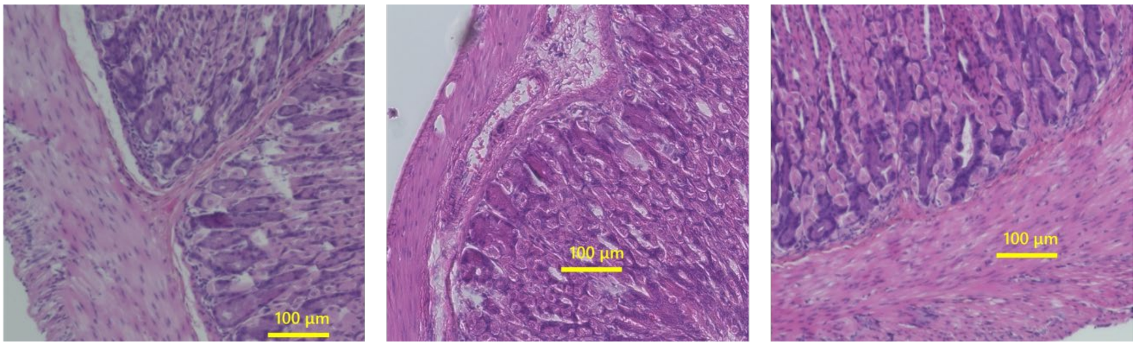
100 µm
100 µm
100 µm

H

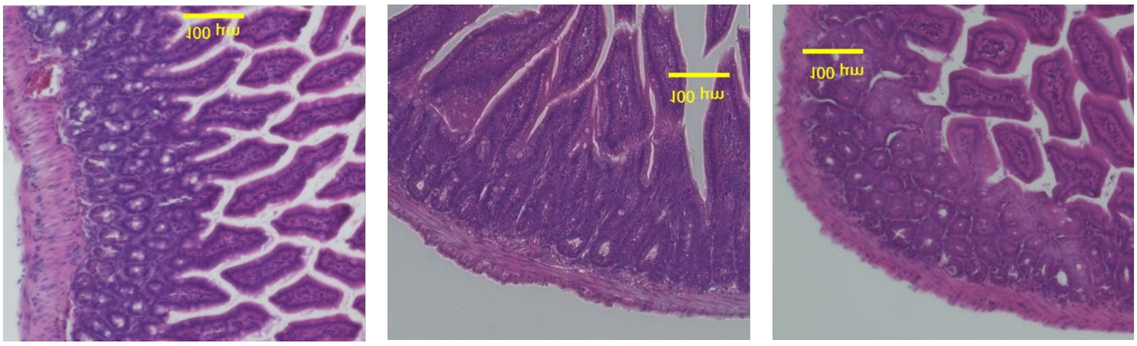

I

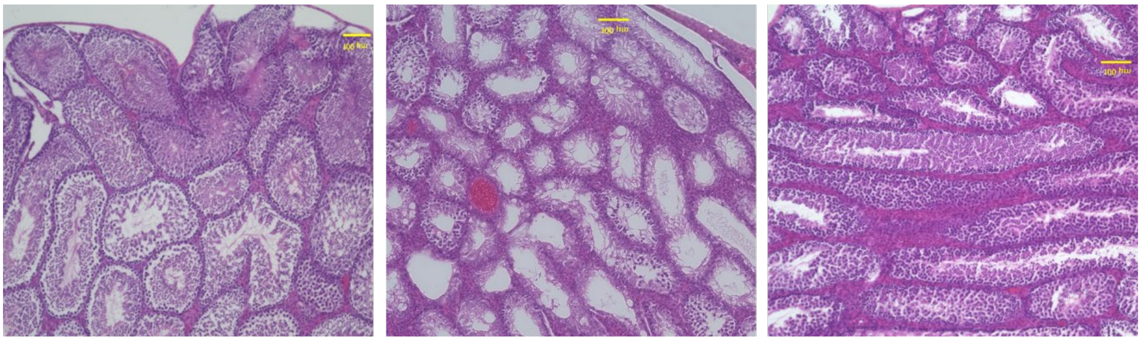

J

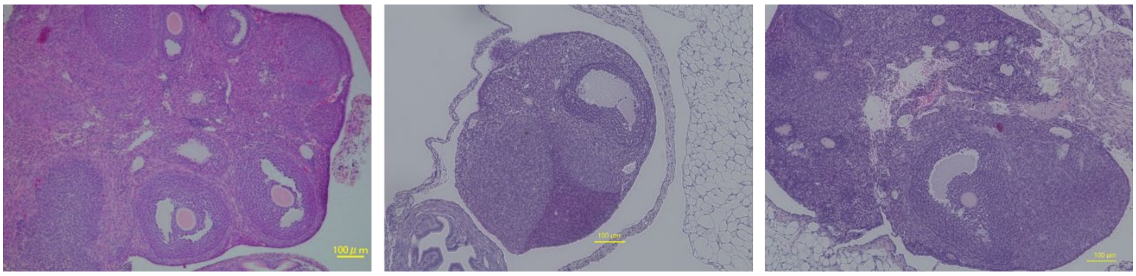

One week after saline administration or four weeks after [^{211}At]AuNP@PEG/RGD or [^{211}At]NaAt administration, the mesentery (A), diaphragm (B), liver (C), kidney (D), spleen (E), pancreas (F), stomach (G), small intestine (H), testis (I), and ovary (J) were harvested and histologically evaluated by HE staining. Leftmost, middle, and rightmost, organ specimens of mice indicate saline, [^{211}At]NaAt, and [^{211}At]AuNP@PEG/RGD

treatment, respectively.

NaAt: [^{211}At]NaAt, AuNP: [^{211}At]AuNP@PEG/RGD
